# Neurological complications after first dose of COVID-19 vaccines and SARS-CoV-2 infection

**DOI:** 10.1038/s41591-021-01556-7

**Published:** 2021-10-25

**Authors:** Martina Patone, Lahiru Handunnetthi, Defne Saatci, Jiafeng Pan, Srinivasa Vittal Katikireddi, Saif Razvi, David Hunt, Xue W. Mei, Sharon Dixon, Francesco Zaccardi, Kamlesh Khunti, Peter Watkinson, Carol A. C. Coupland, James Doidge, David A. Harrison, Rommel Ravanan, Aziz Sheikh, Chris Robertson, Julia Hippisley-Cox

**Affiliations:** 1 Nuffield Department of Primary Health Care Sciences, University of Oxford, Oxford, UK; 2 Nuffield Department of Clinical Neurosciences, University of Oxford, Oxford, UK; 3 Wellcome Centre for Human Genetics, University of Oxford, Oxford, UK; 4 Department of Mathematics and Statistics, University of Strathclyde, Glasgow, UK; 5 MRC/CSO Social and Public Health Sciences Unit, University of Glasgow, Glasgow, UK; 6 Institute of Neurological Sciences, Glasgow, UK; 7 Centre for Clinical Brain Sciences and UK Dementia Research Institute, University of Edinburgh, Edinburgh, UK; 8 Leicester Real World Evidence Unit, Leicester Diabetes Centre, University of Leicester, Leicester, UK; 9 NIHR Biomedical Research Centre, Oxford University Hospitals NHS Trust, Oxford, UK; 10 Division of Primary Care, School of Medicine, University of Nottingham, Nottingham, UK; 11 Intensive Care National Audit and Research Centre, London, UK; 12 London School of Hygiene and Tropical Medicine, London, UK; 13 NHS Blood and Transplant, Bristol, UK; 14 Usher Institute, University of Edinburgh, Edinburgh, UK; 15 Public Health Scotland, Glasgow, UK

**Keywords:** Neuroscience, Neurological disorders

## Abstract

Emerging reports of rare neurological complications associated with COVID-19 infection and vaccinations are leading to regulatory, clinical and public health concerns. We undertook a self-controlled case series study to investigate hospital admissions from neurological complications in the 28 days after a first dose of ChAdOx1nCoV-19 (*n* = 20,417,752) or BNT162b2 (*n* = 12,134,782), and after a SARS-CoV-2-positive test (*n* = 2,005,280). There was an increased risk of Guillain–Barré syndrome (incidence rate ratio (IRR), 2.90; 95% confidence interval (CI): 2.15–3.92 at 15–21 days after vaccination) and Bell’s palsy (IRR, 1.29; 95% CI: 1.08–1.56 at 15–21 days) with ChAdOx1nCoV-19. There was an increased risk of hemorrhagic stroke (IRR, 1.38; 95% CI: 1.12–1.71 at 15–21 days) with BNT162b2. An independent Scottish cohort provided further support for the association between ChAdOx1nCoV and Guillain–Barré syndrome (IRR, 2.32; 95% CI: 1.08–5.02 at 1–28 days). There was a substantially higher risk of all neurological outcomes in the 28 days after a positive SARS-CoV-2 test including Guillain–Barré syndrome (IRR, 5.25; 95% CI: 3.00–9.18). Overall, we estimated 38 excess cases of Guillain–Barré syndrome per 10 million people receiving ChAdOx1nCoV-19 and 145 excess cases per 10 million people after a positive SARS-CoV-2 test. In summary, although we find an increased risk of neurological complications in those who received COVID-19 vaccines, the risk of these complications is greater following a positive SARS-CoV-2 test.

## Abstract (web summary)

A self-controlled case series analysis of nearly 32 million people in England shows an increased risk of rare neurological complications in those who received COVID-19 vaccines and following SARS-CoV-2 infection. The results highlight 38 excess cases of Guillain–Barré syndrome per 10 million ChAdOx1nCoV-19 vaccinations.

## Main

The coronavirus disease 19 (COVID-19) pandemic has seen the development and deployment of vaccines at an unprecedented speed and scale. Several vaccines including ChAdOx1nCoV-19 and BNT162b2 are approved for use in multiple countries and these have been shown to reduce COVID-19 infections, transmissions, hospitalizations and deaths in randomized controlled trials and real-world effectiveness studies^1–6^. However, the clinical trials were underpowered to detect rare adverse events^3,4^ that are important for ongoing risk–benefit evaluations of these vaccines and for informing post-vaccination clinical practice. Therefore, the identification of such rare adverse events is now a global scientific priority.

The increased risk of cerebral venous sinus thrombosis following the ChAdOx1nCoV-19 vaccine is an example of a rare adverse neurological event^7,8^. These findings have prompted the United Kingdom, several European countries and two Canadian provinces to limit the use of the ChAdOx1nCoV-19 vaccine or restrict its use pending further risk–benefit analysis in those at low risk of severe outcomes from infection. Furthermore, two cases of transverse myelitis were identified in the treatment arm during ChAdOx1nCoV-19 clinical trials^3^. These cases triggered two temporary study pauses, including careful regulatory review by the UK’s Medicines and Healthcare Products Regulatory Agency (MHRA). One case of transverse myelitis was considered to be possibly causally related to the vaccine, and an association of rare neuroinflammatory side-effects with ChAdOx1nCoV-19 could not be ruled out.

Since the start of large-scale vaccine programs across the world, additional case reports have linked other neurological adverse events to COVID-19 vaccination, including Guillain–Barré syndrome^9–11^. Furthermore, surveillance studies have found a possible link between severe acute respiratory syndrome coronavirus 2 (SARS-CoV-2) infection and neurological events, including Guillain–Barré syndrome and myelitis^12,13^. However, case reports and surveillance studies are limited by small numbers, as well as potential selection and recording biases. Therefore, detailed assessments of potential neurological adverse events associated with COVID-19 vaccines and infection are urgently needed.

The English National Immunisation (NIMS) Database of COVID-19 vaccination includes data on vaccine type, date and doses for all people vaccinated in England. We linked NIMS, at the individual patient level, to national data for mortality, hospital admissions and SARS-CoV-2 infection data to examine the associations between the first dose of ChAdOx1nCoV-19 or BNT162b2 vaccines and neurological complications: acute central nervous system (CNS) demyelinating events, encephalitis meningitis and myelitis, Guillain–Barré syndrome, Bell’s palsy, myasthenic disorders, hemorrhagic stroke and subarachnoid hemorrhage. We use the same population to investigate the associations between a positive SARS-CoV-2 test as a secondary exposure and the same neurological conditions. Incidence rate ratios (IRRs), that is, the rates of hospital admission or death from each neurological outcome in the risk period after vaccination or after a positive test relative to the baseline period, were estimated using the self-controlled case series (SCCS) methodology (Fig. 2)^14,15^. A relative incidence greater than 1 indicates an increased risk after vaccination or a SARS-CoV-2-positive test. We also carried out an independent replication of the risk of neurological outcomes in a national cohort from Scotland using the SCCS design.

## Results

Overall, 32,552,534 people received their first dose of COVID19 vaccine (ChAdOx1nCoV-19, *n* = 20,417,752; BNT162b2, *n* = 12,134,782) in England between 1 December 2020 and 31 May 2021. In the population of vaccinated people, 2,005,280 (~6%) had a SARS-CoV-2-positive test. Of those with a positive test, 1,838,628 (~91%) had their SARS-CoV-2 test prior to vaccination. The baseline characteristics of the study population are listed in Table 1.

**Table 1:** Baseline demographic characteristics of people receiving either ChAdOx1nCoV-19 or BNT162b2 vaccines or testing positive to SARS-CoV-2 virus (in those vaccinated with either vaccine), in England between 1 December 2020 and 31 May 2021

	ChAdOx1nCoV-19 vaccine	BNT162b2 mRNA vaccine	Positive SARS-CoV-2 test (in those vaccinated)
	% (*n*)	% (*n*)	% (*n*)
Total number of people	20,417,752	12,134,782	2,005,280
**Sex**			
Female	49.7 (10,150,568)	54.5 (6,608,730)	55.5 (1,113,100)
Male	45.3 (9,240,726)	41.0 (4,978,327)	40.6 (814,383)
Sex not recorded	5.0 (1,026,458)	4.5 (547,725)	3.9 (77,797)
**Age groups (years)**			
Mean (s.d.)	55.1 (14.8)	55.9 (20.1)	50.1 (16.9)
16–29	5.2 (1,063,302)	8.7 (1,061,130)	11.3 (226,312)
30–39	7.9 (1,610,998)	21.4 (2,598,709)	17.1 (342,943)
40–49	21.8 (4,442,963)	11.5 (1,399,336)	21.9 (438,788)
50–59	27.4 (5,593,753)	12.5 (1,521,131)	23.9 (479,082)
60–69	19.8 (4,042,273)	14.1 (1,714,392)	12.9 (259,429)
70–79	13.7 (2,803,106)	16.0 (1,942,863)	6.4 (128,302)
80–89	3.2 (646,397)	13.4 (1,620,062)	4.5 (90,947)
90+	1.1 (214,960)	2.3 (277,159)	2.0 (39,477)
**Ethnicity**			
White	72.4 (14,777,834)	74.1 (8,986,385)	70.0 (1,402,843)
Indian	2.2 (448,696)	2.7 (324,499)	4.0 (80,762)
Pakistani	1.3 (274,143)	1.3 (155,524)	3.0 (61,018)
Bangladeshi	0.6 (117,396)	0.4 (53,690)	1.1 (22,900)
Other Asian	1.0 (207,130)	1.2 (145,342)	1.8 (35,373)
Black Caribbean	0.6 (124,735)	0.5 (61,242)	0.7 (13,789)
Black African	1.1 (228,758)	1.0 (121,497)	1.6 (32,185)
Chinese	0.4 (75,432)	0.4 (45,604)	0.2 (4,374)
Other ethnic group	1.9 (387,650)	1.9 (231,506)	2.6 (51,763)
Ethnicity not recorded	18.5 (3,775,978)	16.6 (2,009,493)	15.0 (300,273)
**Neurological history**			
Acute CNS demyelinating events	<0.1 (3,628)	<0.1 (2,028)	<0.1 (463)
Prior encephalitis, meningitis and myelitis	<0.1 (4,322)	<0.1 (2,431)	<0.1 (855)
Prior Guillain–Barré syndrome	<0.1 (1,855)	<0.1 (1,226)	<0.1 (294)
Prior Bell’s palsy	<0.1 (9,275)	0.1 (6,200)	0.1 (1,396)
Prior myasthenic disorder	<0.1 (3,129)	<0.1 (2,609)	<0.1 (461)
Prior hemorrhagic stroke	<0.1 (6,517)	<0.1 (4,064)	0.1 (1,318)
Prior subarachnoid hemorrhage	<0.1 (5,350)	<0.1 (2,776)	<0.1 (822)

**Fig. 1:**
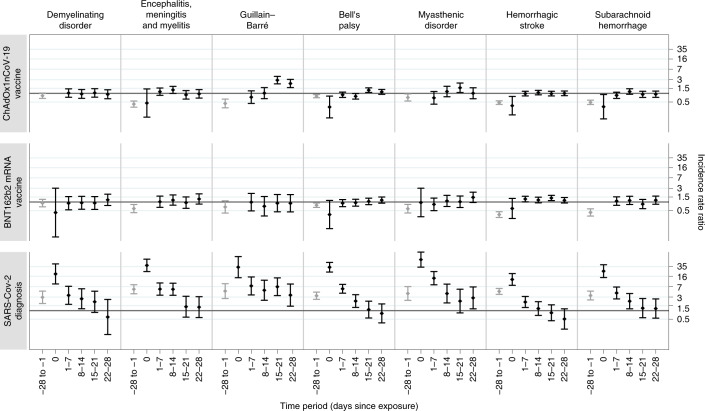
IRRs and 95% CIs for neurological outcomes following ChAdOx1nCoV-19 vaccination, BNT162b2 vaccination and positive SARS-CoV-2 test. The IRRs are presented for pre-defined risk periods (0, 1–7, 8–14, 15–21 and 22–28 days) after each exposure and for the pre-risk period (28 days prior to exposure) and computed using a population of *n* = 32,553,534 vaccinated individuals. The horizontal bold line indicates an IRR of 1.

The results for the investigated neurological outcomes following each exposure (ChAdOx1nCoV-19, BNT162b2 and positive SARS-CoV-2 test) are given for pre-specified time periods: 0, 1–7, 8–14, 15–21 and 22–28 days, as well as collectively for the 1–28 day period. The baseline characteristics of people who had these neurological outcomes are listed in Table 2. The IRRs for each outcome in the risk periods immediately before and after each exposure are given in Table 3 and Fig. 1.

**Table 2:** Demographic characteristics of patients who had the individual outcomes in the 28 days following a COVID-19 vaccine first dose or SARS-CoV-2 infection in the vaccinated population in England from 1 December 2020 to 31 May 2021

	Acute CNS demyelinating events			Encephalitis, meningitis and myelitis			Guillain–Barré		
	ChAdOx1nCoV-19 vaccine	BNT162b2 mRNA vaccine	Positive SARS-CoV-2 test	ChAdOx1nCoV-19 vaccine	BNT162b2 mRNA vaccine	Positive SARS-CoV-2 test	ChAdOx1nCoV-19 vaccine	BNT162b2 mRNA vaccine	Positive SARS-CoV-2 test
	% (*n*)	% (*n*)	% (*n*)	% (*n*)	% (*n*)	% (*n*)	% (*n*)	% (*n*)	% (*n*)
Total number of people	144	69	31	188	97	83	153	34	43
Women	57.6 (83)	65.2 (45)	54.8 (17)	58.5 (110)	56.7 (55)	45.8 (38)	45.1 (69)	44.1 (15)	46.5 (20)
Men	42.4 (61)	34.8 (24)	45.2 (14)	41.5 (78)	43.3 (42)	54.2 (45)	54.9 (84)	55.9 (19)	53.5 (23)
Age groups (years)									
Mean (s.d.)	55.6 (15.8)	59.0 (15.3)	59.9 (18.9)	58.9 (16.9)	66.8 (19.1)	57.4 (15.5)	60.6 (13.5)	67.0 (17.1)	60.0 (15.0)
16–29	7.6 (11)	*		4.3 (8)	6.2 (6)	*	*	*	
30–39	10.4 (15)	*	16.1 (5)	12.8 (24)	5.2 (5)	10.8 (9)	6.5 (10)	0	*
40–49	13.2 (19)	13.0 (9)	22.6 (7)	12.2 (23)	6.2 (6)	14.5 (12)	11.1 (17)	*	16.3 (7)
50–59	25.0 (36)	24.6 (17)	*	18.6 (35)	15.5 (15)	25.3 (21)	24.8 (38)	*	23.3 (10)
60–69	25.0 (36)	31.9 (22)	16.1 (5)	20.7 (39)	12.4 (12)	20.5 (17)	30.1 (46)	17.6 (6)	27.9 (12)
70–79	15.3 (22)	7.2 (5)	19.4 (6)	19.1 (36)	22.7 (22)	18.1 (15)	19.6 (30)	35.3 (12)	11.6 (5)
80–89	*	13.0 (9)	16.1 (5)	10.1 (19)	26.8 (26)	6.0 (5)	5.2 (8)	14.7 (5)	*
90+	*	*	*	*	5.2 (5)	*	*	*	*
Positive SARS-CoV-2 test									
Before vaccination	10.4 (15)	8.7 (6)	–	6.9 (13)	5.1 (5)	**–**	5.2 (8)	*	**–**
After vaccination	*	7.2 (5)	–	6.9 (13)	10.3 (10)	**–**	*	*	**–**

	Bell’s palsy			Myasthenic disorder			Hemorrhagic stroke		
	ChAdOx1nCoV-19 vaccine	BNT162b2 mRNA vaccine	Positive SARS-CoV-2 test	ChAdOx1nCoV-19 vaccine	BNT162b2 mRNA vaccine	Positive SARS-CoV-2 test	ChAdOx1nCoV-19 vaccine	BNT162b2 mRNA vaccine	Positive SARS-CoV-2 test
	% (*n*)	% (*n*)	% (*n*)	% (*n*)	% (*n*)	% (*n*)	% (*n*)	% (*n*)	% (*n*)
Total number of people	435	250	112	99	94	67	533	385	73
Women	52.4 (228)	55.2 (138)	42.9 (48)	45.5 (45)	37.2 (35)	29.9 (20)	55.2 (294)	55.1 (212)	47.9 (35)
Men	47.6 (207)	44.8 (112)	57.1 (64)	54.5 (54)	62.8 (59)	70.1 (47)	44.8 (239)	44.9 (173)	52.1 (38)
Age groups (years)									
Mean (s.d.)	62.6 (15.5)	70.8 (15.2)	67.1 (15.8)	67.2 (15.2)	72.7 (15.8)	66.8 (14.3)	70.9 (14.4)	77.5 (11.7)	69.0 (17.5)
16–29	2.5 (11)	*	*	*	*	*	1.5 (8)	*	*
30–39	5.3 (23)	2.0 (5)	4.5 (5)	*	*	*	1.1 (6)	1.3 (5)	*
40–49	9.2 (40)	4.4 (11)	4.5 (5)	6.1 (6)	*	*	4.5 (24)	1.3 (5)	12.3 (9)
50–59	26.9 (117)	16.8 (42)	18.8 (21)	13.1 (13)	7.4 (7)	13.4 (9)	14.4 (77)	4.9 (19)	8.2 (6)
60–69	20.7 (90)	16.8 (42)	27.7 (31)	28.3 (28)	13.8 (13)	29.9 (20)	19.5 (104)	11.2 (43)	11.0 (8)
70–79	20.7 (90)	20.8 (52)	18.8 (21)	27.3 (27)	27.7 (26)	28.4 (19)	30.6 (163)	28.6 (110)	28.8 (21)
80–89	10.6 (46)	30.0 (75)	17.9 (20)	17.2 (17)	35.1 (33)	13.4 (9)	20.8 (111)	41.0 (158)	23.3 (17)
90+	4.1 (18)	7.6 (19)	6.3 (7)	*	6.4 (6)	*	7.5 (40)	11.4 (44)	9.6 (7)
Positive SARS-CoV-2 test									
Before vaccination	8.7 (38)	7.2 (18)	–	7.1 (7)	*	–	8.1 (43)	3.7 (14)	–
After vaccination	3.2 (14)	4.4 (11)	–	*	11.7 (11)	–	3.6 (19)	8.31 (32)	–

	Subarachnoid hemorrhage		
	ChAdOx1nCoV-19 vaccine	BNT162b2 mRNA vaccine	Positive SARS-CoV-2 test
	% (*n*)	% (*n*)	% (*n*)
Total number of people	308	151	68
Women	54.5 (168)	62.9 (95)	50.1 (34)
Men	45.1 (139)	36.4 (55)	50.1 (34)
Not recorded	0.3 (1)	0.7 (1)	0
Age (years)			
Mean (s.d.)	63.5 (15.5)	69.7 (14.2)	64.5 (17.2)
16–29	2.6 (8)	*	*
30–39	3.9 (12)	3.3 (5)	8.8 (6)
40–49	9.1 (28)	*	11.8 (8)
50–59	25.6 (79)	17.2 (26)	17.2 (26)
60–69	23.7 (73)	19.2 (29)	19.2 (29)
70–79	18.2 (56)	27.8 (42)	26.5 (18)
80–89	12.7 (39)	25.8 (39)	14.7 (10)
90+	4.2 (13)	*	*
Positive SARS-CoV-2 test			
Before vaccination	9.1 (28)	6.6 (10)	
After vaccination	3.6 (11)	6.0 (9)	

*Cells with <5 are suppressed.

**Table 3:** IRR and 95% CI for individual outcomes in pre-defined risk periods immediately before and after exposure to vaccination and before and after a positive SARS-CoV-2 test result, adjusted for calendar time from 1 December 2020 to 31 May 2021

Time period	ChAdOx1nCoV-19 vaccine		BNT162b2 mRNA vaccine		Positive SARS-CoV-2 test	
	Events	IRR (95% CI)	Events	IRR (95% CI)	Events	IRR (95% CI)
Acute CNS demyelinating events						
Baseline	473	1.00	244	1.00	53	1.00
−28 to −1 days	117	0.83 (0.67–1.03)	58	0.92 (0.68–1.24)	27	2.94 (1.79–4.83)
Day 0	0	NA	*	0.43 (0.16–3.05)	7	19.34 (8.63–43.38)
1–7 days	38	1.03 (0.73–1.44)	15	0.91 (0.53–1.55)	9	3.45 (1.67–7.14)
8–14 days	35	0.95 (0.67–1.36)	16	0.94 (0.56–1.58)	7	2.61 (1.17–5.84)
15–21 days	38	1.04 (0.74–1.46)	16	0.92 (0.55–1.55)	6	2.05 (0.87–4.82)
22–28 days	33	0.93 (0.65–1.33)	21	1.19 (0.75–1.89)	*	0.60 (0.15–2.47)
1–28 days	144	0.97 (0.78–1.22)	68	1.02 (0.75–1.40)	*	1.67 (0.93–3.00)
Encephalitis, meningitis and myelitis						
Baseline	602	1.00	275	1.00	66	1.00
−28 to −1 days	78	0.42 (0.33–0.54)	45	0.59 (0.42–0.82)	78	5.64 (3.91–8.13)
Day 0	*	0.46 (0.15–1.43)	0	NA	23	38.57 (23.41–63.56)
1–7 days	52	1.15 (0.85–1.54)	22	1.02 (0.65–1.61)	24	5.71 (3.49–9.32)
8–14 days	58	1.32 (0.99–1.76)	27	1.17 (0.77–1.77)	24	5.63 (3.46–9.15)
15–21 days	37	0.89 (0.63–1.25)	21	0.95 (0.60–1.50)	6	1.38 (0.59–3.21)
22–28 days	38	0.96 (0.68–1.35)	27	1.28 (0.85–1.93)	6	1.33 (0.57–3.08)
1–28 days	185	1.07 (0.87–1.31)	97	1.14 (0.86–1.51)	60	2.70 (1.78–4.11)
Guillain–Barré syndrome						
Baseline	264	1.00	109	1.00	30	1.00
−28 to −1 days	41	0.44 (0.31–0.63)	21	0.67 (0.41–1.10)	23	4.85 (2.67–8.82)
Day 0	0	NA	0	NA	7	33.37 (14.21–78.36)
1–7 days	17	0.74 (0.44–1.23)	9	0.99 (0.49–2.00)	11	7.36 (3.57–15.18)
8–14 days	23	1.02 (0.65–1.59)	7	0.71 (0.32–1.56)	8	5.19 (2.31–11.65)
15–21 days	65	2.90 (2.15–3.92)	9	0.91 (0.45–1.84)	11	6.89 (3.37–14.09)
22–28 days	48	2.21 (1.59–3.09)	9	0.90 (0.45–1.82)	6	3.51 (1.44–8.57)
1–28 days	153	2.04 (1.60–2.60)	34	0.86 (0.54–1.36)	36	5.25 (3.00–9.18)
Bell’s palsy						
Baseline	1,356	1.00	712	1.00	134	1.00
−28 to −1 days	328	0.80 (0.70–0.91)	168	0.77 (0.65–0.92)	86	3.34 (2.49–4.48)
Day 0	5	0.33 (0.14–0.80)	*	0.36 (0.12–1.13)	35	33.23 (22.57–48.94)
1–7 days	95	0.89 (0.72–1.11)	54	0.91 (0.69–1.21)	44	5.84 (4.09–8.33)
8–14 days	84	0.79 (0.63–0.99)	57	0.95 (0.72–1.25)	17	2.17 (1.30–3.63)
15–21 days	136	1.29 (1.08–1.56)	65	1.05 (0.81–1.37)	9	1.09 (0.55–2.15)
22–28 days	115	1.13 (0.92–1.37)	71	1.17 (0.91–1.50)	7	0.80 (0.37–1.72)
1–28 days	430	1.07 (0.94–1.21)	247	1.06 (0.90–1.26)	77	1.34 (0.91–1.97)
Myasthenic disorder						
Baseline	311	1.00	213	1.00	32	1.00
−28 to −1 days	70	0.72 (0.54–0.96)	44	0.58 (0.41–0.83)	26	4.00 (2.30–6.96)
Day 0	0	NA	*	0.96 (0.30–3.04)	18	61.32 (33.43–112.50)
1–7 days	17	0.70 (0.42–1.16)	18	0.83 (0.50–1.37)	29	13.74 (8.07–23.41)
8–14 days	26	1.16 (0.76–1.77)	24	1.09 (0.70–1.71)	9	3.98 (1.86–8.52)
15–21 days	33	1.57 (1.07–2.30)	22	1.02 (0.64–1.63)	5	2.17 (0.84–5.63)
22–28 days	23	1.01 (0.65–1.57)	27	1.46 (0.96–2.22)	6	2.81 (1.16–6.79)
1–28 days	99	1.23 (0.94–1.62)	91	1.18 (0.88–1.59)	49	3.01 (1.70–5.36)
Hemorrhagic stroke						
Baseline	1,456	1.00	811	1.00	173	1.00
−28 to −1 days	232	0.47 (0.41–0.55)	86	0.36 (0.29–0.46)	163	4.73 (3.72–6.02)
Day 0	7	0.37 (0.18–0.78)	6	0.59 (0.27–1.33)	20	12.42 (7.73–19.95)
1–7 days	131	0.98 (0.82–1.18)	93	1.27 (1.02–1.59)	23	2.01 (1.29–3.15)
8–14 days	141	1.06 (0.89–1.27)	89	1.18 (0.94–1.48)	14	1.19 (0.69–2.07)
15–21 days	128	0.98 (0.81–1.18)	106	1.38 (1.12–1.71)	10	0.84 (0.44–1.61)
22–28 days	126	1.00 (0.83–1.21)	91	1.15 (0.92–1.44)	6	0.51 (0.23–1.16)
1–28 days	526	1.02 (0.90–1.15)	379	1.24 (1.07–1.43)	53	0.85 (0.57–1.26)
Subarachnoid hemorrhage						
Baseline	971	1.00	415	1.00	88	1.00
−28 to −1 days	157	0.49 (0.41–0.58)	53	0.43 (0.32–0.58)	60	3.40 (2.38–4.86)
Day 0	*	0.34 (0.13–0.91)	0	NA	19	24.22 (14.50–40.45)
1–7 days	70	0.85 (0.66–1.10)	38	1.08 (0.77–1.52)	23	4.17 (2.59–6.71)
8–14 days	93	1.16 (0.93–1.44)	41	1.14 (0.82–1.59)	12	2.15 (1.16–3.99)
15–21 days	72	0.92 (0.72–1.18)	31	0.85 (0.58–1.23)	7	1.23 (0.57–2.67)
22–28 days	69	0.94 (0.73–1.20)	41	1.16 (0.84–1.61)	7	1.19 (0.55–2.58)
1–28 days	304	1.01 (0.86–1.18)	151	1.05 (0.84–1.30)	49	1.51 (0.96–2.36)

*Cells with <5 are suppressed.

NA, not applicable.

### Neurological outcomes

#### Acute CNS demyelinating events

A total of 1,105 people had a hospital admission with an acute CNS demyelinating event. Of these, 131 (11.9%) had a positive SARS-CoV-2 test including 121 (10.9%) who had a positive test prior to vaccination. There were fewer than five deaths overall. There was no association between the ChAdOx1nCoV-19 or BNT162b2 vaccination and admission with an acute CNS demyelinating event in any of the pre-defined 7 day risk periods. There was an increased risk of hospital admission or death for acute CNS demyelinating events on the day of a positive SARS-CoV-2 test (IRR, 19.34; 95% CI: 8.63–43.38), and 1–7 days (IRR, 3.45; 95% CI: 1.67–7.14) and 8–14 days (IRR, 2.61; 95% CI: 1.17–5.84) after a positive test. In the 1–28 days post-exposure period we did not observe any association with ChAdOx1nCoV-19 (IRR, 0.97; 95% CI: 0.78–1.22) or with BNT162b2 (IRR, 1.02; 95% CI: 0.75–1.40), but a possible association with SARS-CoV-2 infection was observed (IRR, 1.67; 95% CI: 0.93–3.00).

#### Encephalitis, meningitis and myelitis

Encephalitis-, meningitis- and myelitis-related admissions occurred in 1,285 people. Of these, 255 (19.8%) had a SARS-CoV-2-positive test including 213 (16.6%) who had a positive test prior to vaccination. There were 39 deaths (in six of which the SARS-CoV-2 test was positive). There was a trend towards increased risk of encephalitis, meningitis and myelitis after ChAdOx1nCoV-19 vaccination (IRR, 1.32; 95% CI: 0.99–1.76 at 8–14 days) and no association with the BNT162b2 vaccine in any of the 7 day risk periods. There was an increased risk of hospital admission or death for this outcome on the day of a SARS-CoV-2-positive test (IRR, 38.57; 95% CI: 23.41–63.56), and 1–7 days (IRR, 5.71; 95% CI: 3.49–9.32) and 8–14 days (IRR, 5.63; 95% CI: 3.64–9.15) after the positive test. In the 1–28 days post-exposure period we did not observe an association with ChAdOx1nCoV-19 (IRR, 1.07; 95% CI: 0.87–1.31) or with BNT162b2 (IRR, 1.14; 95% CI: 0.86–1.51), but an association with SARS-CoV-2 infection was identified (IRR, 2.07; 95% CI: 1.78–4.11).

#### Guillain–Barré syndrome

Guillain–Barré syndrome-related admissions occurred in 622 people. Of these, 110 (17.7%) had a positive SARS-CoV-2 test including 99 (15.9%) who had a positive test prior to vaccination. There were 11 deaths (SARS-CoV-2 test was not positive in any of these deaths). There was an increased risk of hospital admission or death for Guillain–Barré syndrome at 15–21 days (IRR, 2.90; 95% CI: 2.15–3.92) and 22–28 days (IRR, 2.21; 95% CI: 1.59–3.09) following the first dose of ChAdOx1nCoV-19. No association was found with the BNT162b2 vaccine. There was an increased risk of Guillain–Barré syndrome following a positive SARS-CoV-2 test (IRR, 33.37; 95% CI: 14.21–78.36 at day 0; IRR, 7.36; 95% CI: 3.57–15.18 at 1–7 days; IRR, 5.19; 95% CI: 2.31–11.65 at 8–14 days; IRR, 6.89; 95% CI: 3.37–14.09 at 15–22 days; IRR, 3.51; 95% CI: 1.44–8.57 at 22–28 days). In the 1–28 days post-vaccination period we found an increased risk of Guillain–Barré syndrome after ChAdOx1nCoV-19 vaccination (IRR, 2.04; 95% CI: 1.60–2.60) but not after BNT162b2 vaccination (IRR, 0.86; 95% CI: 0.54–1.36). Also, an increased risk of Guillain–Barré syndrome was observed in the 1–28 days period after a SARS-CoV-2-positive test (IRR, 5.25; 95% CI: 3.00–9.18).

#### Bell’s palsy

There were 3,249 people admitted to hospital with Bell’s palsy. Of these, 391 (12.0%) had a positive SARS-CoV-2 test including 334 (10.3%) who had a positive test prior to vaccination. There were no deaths. There was an increased risk of hospital admission for Bell’s palsy at 15–21 days after the first dose of ChAdOx1nCoV-19 (IRR, 1.29; 95% CI: 1.08–1.56). No association was found with the BNT162b2 vaccine. There was an increased risk of Bell’s palsy following a positive SARS-CoV-2 test (IRR, 33.23; 95% CI: 22.57–48.94 at day 0; IRR, 5.84; 95% CI: 4.09–8.33 at 1–7 days; IRR, 2.17; 95% CI: 1.30–3.63 at 8–14 days). In the 1–28 days post-exposure period we did not observe any association with ChAdOx1nCoV-19 (IRR, 1.07; 95% CI: 0.94–1.21), BNT162b2 (IRR, 1.06; 95% CI: 0.90–1.26) or SARS-CoV-2 infection (IRR, 1.34; 95% CI: 0.91–1.97). In the cohort of patients with Bell’s palsy, a small proportion (6%) had a suspected concurrent diagnosis of cerebral infarction. We undertook a post-hoc sensitivity analysis excluding these people but the results did not change from the main analysis, as shown in Supplementary Table 1.

#### Myasthenic disorder

There were 831 hospital admissions for myasthenic disorder. Of these, 137 people (16.5%) had a positive SARS-CoV-2 test including 110 (13.2%) who had a positive test prior to vaccination. There were 30 deaths (in 10 of which the SARS-CoV-2 test was positive). There was an increased risk of hospital admission or death for myasthenic disorder at 15–21 days after the first dose of ChAdOx1nCoV-19 (IRR, 1.57; 95% CI: 1.07–2.30). No association was found with the BNT162b2 vaccine. There was an increased risk of hospital admission or death with myasthenic disorder following a positive SARS-CoV-2 test (IRR, 61.32; 95% CI: 33.43–112.50 at day 0; IRR, 13.74; 95% CI: 8.07–23.41 at 1–7 days; IRR, 3.98; 95% CI: 1.86–8.52 at 8–14 days; IRR, 2.81; 95% CI: 1.16–6.79 at 22–28 days). In the 1–28 days post-exposure period we did not observe any association with ChAdOx1nCoV-19 (IRR, 1.23; 95% CI: 0.94–1.62) or BNT162b2 (IRR, 1.18; 95% CI: 0.88–1.59), but an increased risk after a SARS-CoV-2-positive test was identified (IRR, 3.01; 95% CI: 1.70–5.36).

#### Hemorrhagic stroke

There were 3,503 hospital admissions due to hemorrhagic stroke. Of these, 483 people (13.8%) had a positive SARS-CoV-2 test including 392 (11.2%) who had a positive test prior to vaccination. There were 803 deaths (in 83 of which the SARS-CoV-2 test was positive). No association was found with the ChAdOx1nCoV-19 vaccine in any of the pre-specified time periods. There was an increased risk of hospital admission or death from hemorrhagic stroke at 1–7 days (IRR, 1.27; 95% CI: 1.02–1.59) and 15–21 days (IRR, 1.38; 95% CI: 1.12–1.71) after the first dose of BNT162b2. There was an increased risk of hemorrhagic stroke up to 7 days after a positive SARS-CoV-2 test (IRR, 12.42; 95% CI: 7.73–19.95 at day 0; IRR, 2.01; 95% CI: 1.29–3.15 at 1–7 days). In the 1–28 days post-vaccination period we did not observe an association with ChAdOx1nCoV-19 (IRR, 0.91; 95% CI: 0.88–0.94), but a positive association with the BNT162b2 vaccine was observed (IRR, 1.24; 95% CI: 1.07–1.43). No association was found with SARS-CoV-2 infection in the 1–28 day period after a positive test (IRR, 0.85; 95% CI: 0.57–1.26).

#### Subarachnoid hemorrhage

There were 2,055 hospital admissions due to subarachnoid hemorrhage. Of these, 262 people (12.7%) had a positive SARS-CoV-2 test including 220 (10.7%) who had a positive test prior to vaccination. There were of 273 deaths (in 27 of which the SARS-CoV-2 test was positive). There was no association between the ChAdOx1nCoV-19 and BNT162b2 vaccines and subarachnoid hemorrhage. There was an increased risk of hospital admission or death for subarachnoid hemorrhage on the day of a SARS-CoV-2-positive test (IRR, 24.22; 95% CI: 14.50–40.45), and at 1–7 days (IRR, 4.17; 95% CI: 2.59–6.71) and 8–14 days (IRR, 2.15; 95% CI: 1.16–3.99) after the positive test. In the 1–28 days post-exposure period we did not observe any association with ChAdOx1nCoV-19 (IRR, 1.01; 95% CI: 0.86–1.18) or with BNT162b2 (IRR, 1.05; 95% CI: 0.84–1.30), but a potentially increased risk after the SARS-CoV-2 infection (IRR, 1.51; 95% CI: 0.96–2.36) was observed.

#### Guillain–Barré syndrome and Bell’s palsy: composite outcome

Given that facial weakness can be part of the spectrum of Guillain–Barré syndrome, we investigated the risk of hospital admission or death from co-occurrence of Bell’s palsy and Guillain–Barré syndrome in the same individuals before and after ChAdOx1nCoV-19 and BNT162b2 vaccination in the study period. There were 34 patients admitted to hospital for both Bell’s palsy and Guillain–Barré syndrome. Of these patients, 27 were admitted on the same day for both outcomes, while seven were first admitted for Bell’s palsy and subsequently for Guillain–Barré syndrome. We found an increased risk of co-occurrence of Bell’s palsy and Guillain–Barré syndrome in the 15–21 days (IRR, 28.86; 95% CI: 5.45–152.74) and 22–28 days (IRR, 13.35; 95% CI: 2.48–72.92) after ChAdOx1nCoV-19 vaccination (Supplementary Table 2). In the 1–28 days post-vaccination period we found a positive association with the ChAdOx1nCoV-19 vaccine (IRR, 12.66; 95% CI: 2.70–59.21) but not with the BNT162b2 vaccine (Supplementary Table 2). The IRRs associated with SARS-CoV-2 infection were not estimated due to the small number of events in those with a positive SARS-CoV-2 test.

### National Scottish data

In Scotland 1,982,678 people received the first dose of ChAdOx1nCoV-19 vaccine (~10% of those in England) and 1,077,626 people received the first dose of BNT162b2 vaccine (~10% of those in England) between 1 December 2020 and 31 May 2021. Of those vaccinated, 117,554 (~3%) had a SARS-CoV-2-positive test, either prior to or after vaccination. The age distribution of vaccinated patients and the percentage of vaccinated patients having any prior conditions were similar in the Scottish and English populations (Supplementary Table 3a).

The association between ChAdOx1nCoV-19 and Guillain–Barré syndrome for the 1–28 days post-vaccination period was replicated in Scotland (IRR, 2.31; 95% CI: 1.02–5.24), as shown in Supplementary Table 3b. The timing of this increased risk was consistent with the results seen in England: IRR, 2.15 (95% CI: 0.63–7.31) for 8–14 days, 4.79 (95% CI: 1.72–13.38) for 15–21 days and 3.60 (95% CI: 1.11–11.65) for 22–28 days (Supplementary Table 3c). No association between BNT162b2 and hemorrhagic stroke was seen in Scotland (IRR, 0.65; 95% CI: 0.35–1.20) in the 1–28 days period after vaccination (Supplementary Table 3b). The findings for SARS-CoV-2 test positivity were similar to the English data, with an increased risk of neurological outcomes following a positive test, although the estimates were generally imprecise due to the smaller population and lower rates of infection.

### Subgroup analyses by age group and sex

We tested for interactions between vaccination and age group (>50 years and ≤50 years) and sex for the outcomes for which we observed an increased risk after vaccination. Supplementary Table 4a lists the results of the interaction tests. When interaction terms were statistically significant, the IRR for subgroup analyses by age group or sex have been reported in Supplementary Table 4b. There was a significant interaction (*P* = 0.016) between age group and risk of myasthenic disorder associated with the ChAdOx1nCoV-19 vaccine (IRR, 2.44; 95% CI: 1.07–5.55 at 15–21 days) in people aged ≤50 years. The increased risk of hemorrhagic stroke associated with the BNT162b2 vaccine was significantly higher in female participants (*P* = 0.007): the IRR for hemorrhagic stroke in female participants was 1.44 (95% CI: 1.05–1.96) at 1–7 days and 1.84 (95% CI: 1.40–2.42) at 15–21 days after BNT162b2 vaccination compared with 1.13 (95% CI: 0.82–1.57) at 1–7 days and 0.98 (95% CI: 0.70–1.38) at 15–21 days in male participants.

### Excess events due to exposures

We estimated the number of exposures needed for one excess event and the excess number of events per 10 million exposed for each outcome (Supplementary Table 5). For example, with ChAdOx1nCoV-19 there were 38 excess Guillain–Barré syndrome events per 10 million people vaccinated in the 1–28 days period after vaccination. For BNT1262b2, there were 60 extra cases of hemorrhagic stroke per 10 million people vaccinated in the 1–28 days period after vaccination. For SARS-CoV-2 infection, in the 1–28 days period after a positive test there were an estimated 123 extra events of encephalitis meningitis and myelitis and 145 of Guillain–Barré syndrome per 10 million people with a positive test.

### Associations with negative and positive control outcomes

We examined the associations of exposures with celiac disease as a negative control outcome and with anaphylaxis as a positive control outcome. We found no increased risk of celiac disease (negative control) across the pre-specified time periods for the vaccine exposures, but a decreased risk on the day of vaccination. Anaphylaxis (positive control) showed the expected increased risk in the 0–7 days after the first dose for both vaccines, but not in later periods (Supplementary Table 6).

### Sensitivity analyses

**Extended Data Fig. 1:**
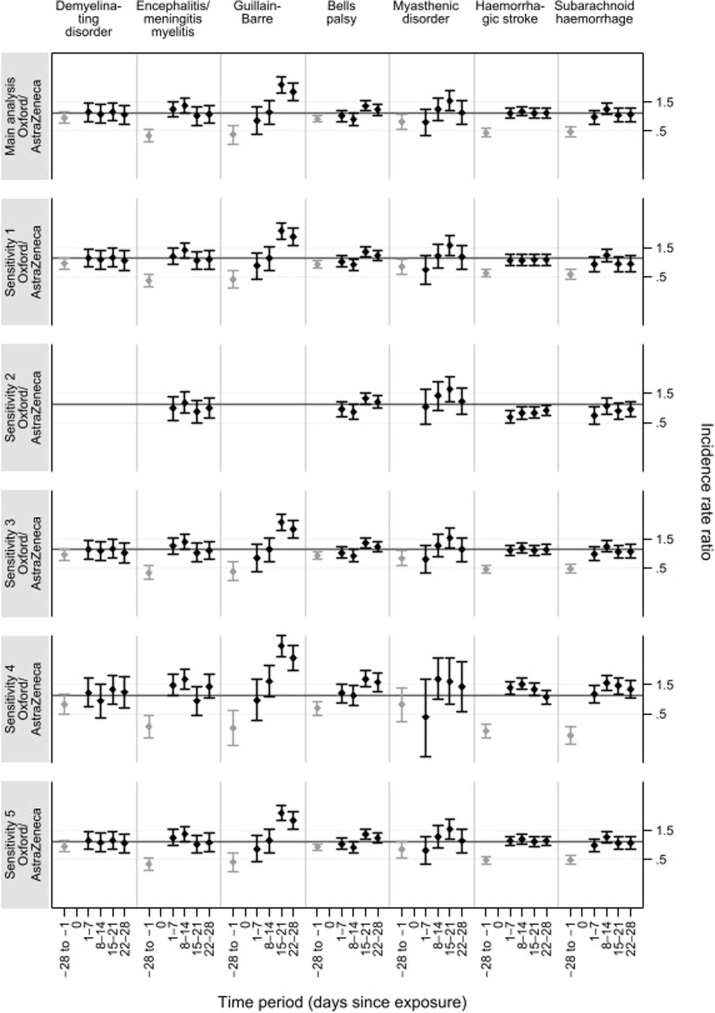
Incidence rate ratios (IRR 95% CI) for single outcomes in pre-defined risk periods immediately before and after exposure to ChAdOx1nCoV-19 vaccine. Incidence rate ratios (IRR 95% CI) for single outcomes in pre-defined risk periods immediately before and after exposure to ChAdOx1nCoV-19 vaccine, adjusted for calendar time from 1 December 2020 to 31 May 2021 computed using a population of size n = 32,553,534 vaccinated individuals. Comparisons between different sensitivity analyses (cells with < 5 are suppressed). Horizontal bold line indicates 1.

**Extended Data Fig. 2:**
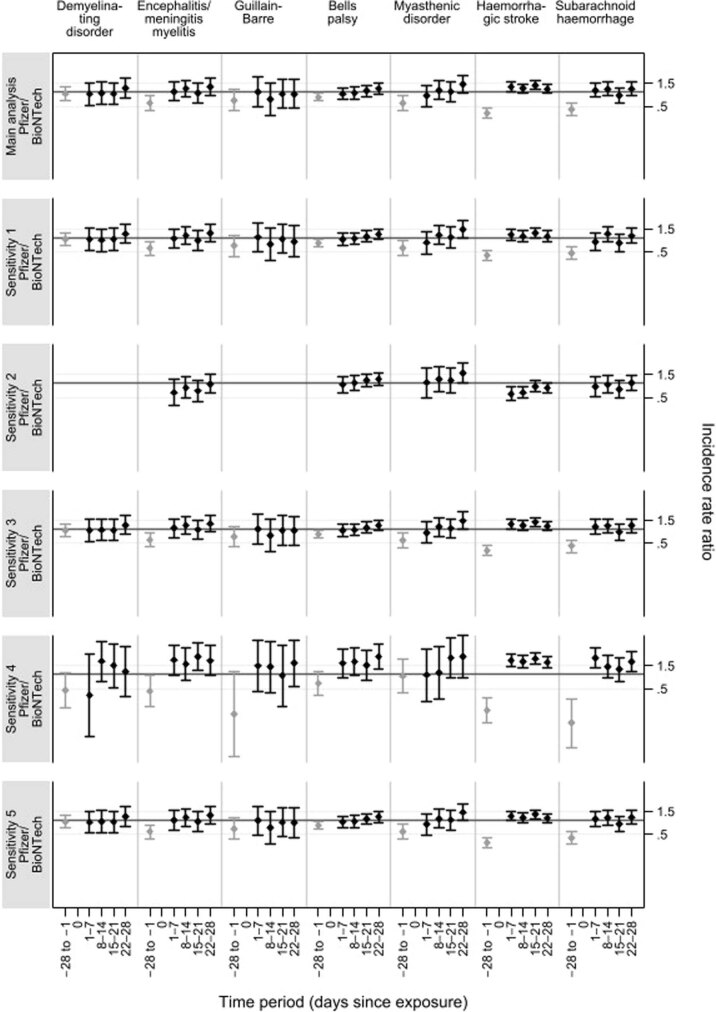
Incidence rate ratios (IRR 95% CI) for single outcomes in pre-defined risk periods immediately before and after exposure to BNT162b2 mRNA vaccine. Incidence rate ratios (IRR 95% CI) for single outcomes in pre-defined risk periods immediately before and after exposure to BNT162b2 mRNA vaccine, adjusted for calendar time from 1 December 2020 to 31 May 2021 computed using a population of size n = 32,553,534 vaccinated individuals. Comparisons between different sensitivity analyses (cells with < 5 are suppressed). Horizontal bold line indicates 1.

We conducted a number of sensitivity analyses to ascertain the robustness of our results. Overall, the main findings were not sensitive to censoring due to death (with the exception of hemorrhagic stroke), removal of patients who had a second dose of the vaccine or adjustment for potential delays in recording. Results for these sensitivity analyses are shown in Supplementary Table 7a and Extended Data Figs. 1 and 2. We also extended the exposure risk period to 5 weeks, to test the suitability of our 28 day risk period and to avoid missing events due to lag in diagnosis. Results were similar to the main analysis (Supplementary Table 7b). An increased risk of Guillain–Barré syndrome was observed in the 29–35 days after ChAdOx1nCoV-19 vaccination (IRR, 1.55; 95% CI: 1.03–2.34).

We have further restricted the analyses to patients who had a positive SARS-CoV-2 test before vaccination. The results did not change from the main analysis for the SARS-CoV-2 test exposure (Supplementary Table 7c), suggesting that there is a higher risk of neurological complications after a positive SARS-CoV-2 test for all outcomes. The number of people infected after vaccination was too small to carry out any additional meaningful analyses. We also restricted the analyses to those who did not have a positive SARS-CoV-2 test. The results did not change from the main analysis for both vaccine exposures (Supplementary Table 7d).

## Discussion

This large population-based study of more than 32 million people investigated the neurological adverse events associated with the ChAdOx1nCoV-19 and BNT162b2 vaccines as well as SARS-CoV-2 infection. We identified several key findings that are timely and of high importance to the public, health policy makers and clinicians across the world. First, we found an increased risk of hospital admission for Guillain–Barré syndrome (15–21 days and 22–28 days), Bell’s palsy (15–21 days) and myasthenic disorders (15–21 days) in those who received the ChAdOx1nCoV-19 vaccine. Second, an increased risk of hospital admission for hemorrhagic stroke (1–7 days and 15–21 days) was observed in those who received the BNT162b2 vaccine. Last, we identified a much greater increase in the risk of neurological outcomes following a positive SARS-CoV-2 test, such as acute CNS demyelinating events, encephalitis meningitis and myelitis, Guillain–Barré syndrome, Bell’s palsy, myasthenic disorders, hemorrhagic stroke and subarachnoid hemorrhage. Apart from hemorrhagic stroke, the results were robust to sensitivity analyses that assessed the assumption that outcomes did not influence subsequent exposures, providing credence to our results.

In those who received the ChAdOx1nCoV-19 vaccine, we estimated 38 excess cases of Guillain–Barré syndrome per 10 million exposed in the 1–28 days risk period. However, we did not observe an increased risk of Guillain–Barré syndrome in those who received the BNT162b2 vaccine. We replicated these findings using an independent national cohort of more than 3 million people from Scotland, which provides strong support for the association between ChAdOx1nCoV-19 vaccine and Guillain–Barré syndrome. The study design and sensitivity analyses addressed some of the key issues related to this association, including confounding through fixed covariates, seasonality and variable exposures to predisposing infections during the pre- and post-vaccination periods. It remains unclear why the ChAdOx1nCoV-19 vaccine appears to contribute to the pathogenesis of Guillain–Barré syndrome while BNT162b2 vaccine does not. Further studies are needed to assess whether antibodies against adenovirus vector-based ChAdOx1nCoV-19 can cross-react with components of the peripheral nerves. Furthermore, we found that Guillain–Barré syndrome and Bell’s palsy co-occur in those who received the ChAdOx1nCoV-19 vaccine. Clinically, this is likely to represent a variant of Guillain–Barré syndrome, in line with the emerging case reports of Guillain–Barré syndrome variants with facial weakness after the ChAdOx1nCoV-19 vaccine^16,17^.

In those who received the BNT162b2 vaccine, we estimated 60 excess cases of hemorrhagic strokes per 10 million exposed in the 28 days after vaccination. We did not observe an increased risk of hemorrhagic stroke in those who received the ChAdOx1nCoV-19 vaccine. However, the magnitude of this association between BNT162b2 and hemorrhagic stroke was reduced in sensitivity analyses that accounted for fatal events. Intriguingly, the increased risk of hemorrhagic stroke associated with BNT162b2 was substantially higher in female participants than in male participants, suggesting that sex may further modify this risk. The mechanisms underlying this disparity in risk between the two vaccines is not clear but several reports have suggested an increased risk of immune thrombocytopenic purpura in individuals receiving mRNA vaccines^18,19^, which in turn could contribute to major bleeding events. It is worth highlighting that the association between the BNT162b2 vaccine and hemorrhagic stroke was not replicated using Scottish data in the context of a low number of events, and thus further validation of this finding is warranted.

In line with previous surveillance studies^14,15^, we identified that all investigated neurological conditions were linked to SARS-CoV-2 infection itself. Specifically, we noted an excess of inflammatory disorders including encephalitis meningitis and myelitis (123 excess cases per 10 million exposed), myasthenic disorders (163 excess cases per 10 million exposed) and Guillain–Barré syndrome (145 excess cases per 10 million exposed) in the 1–28 days period after a positive test. Our post-hoc analysis restricting to those who had a positive test prior to vaccination did not change the results. Unfortunately, the number of people with a SARS-CoV-2-positive test after vaccination was too small to evaluate the risk of neurological complications in this group. The highest IRRs for these neurological outcomes were typically seen on day 0 (the day of the positive test). We included day 0 on its own as a risk period because hospital admission can trigger SARS-CoV-2 testing; however, this could overestimate or underestimate the overall risks associated with infection. The number of people infected in Scotland (3%) was much smaller than in England (~6%), reflecting differences in infection rates and containment measures in the two countries.

Findings from this study have clear clinical and public health implications. Crucially, we found that the risk of neurological complications from infection was substantially higher than the risk of adverse events from vaccinations in our population (for example, 145 excess cases versus 38 excess cases of Guillain–Barré syndrome per 10 million exposed in those who had a positive SARS-CoV-2 test and ChAdOx1nCoV-19 vaccine, respectively). This will need ongoing analysis and monitoring as younger people are vaccinated. Clearly, neurological complications from vaccination and infection reported in this study are rare. However, these rare complications can cause lifelong disability requiring long-term care. Collectively, these results provide timely and valuable information that can help to inform clinical decision making, including facilitating earlier diagnosis, as well as resource allocation for health-care provision. This may be particularly relevant for intensive care unit resource planning, given the potential need for prolonged admission to intensive care units for a proportion of patients with Guillain–Barré syndrome^20^.

**Fig. 2:**
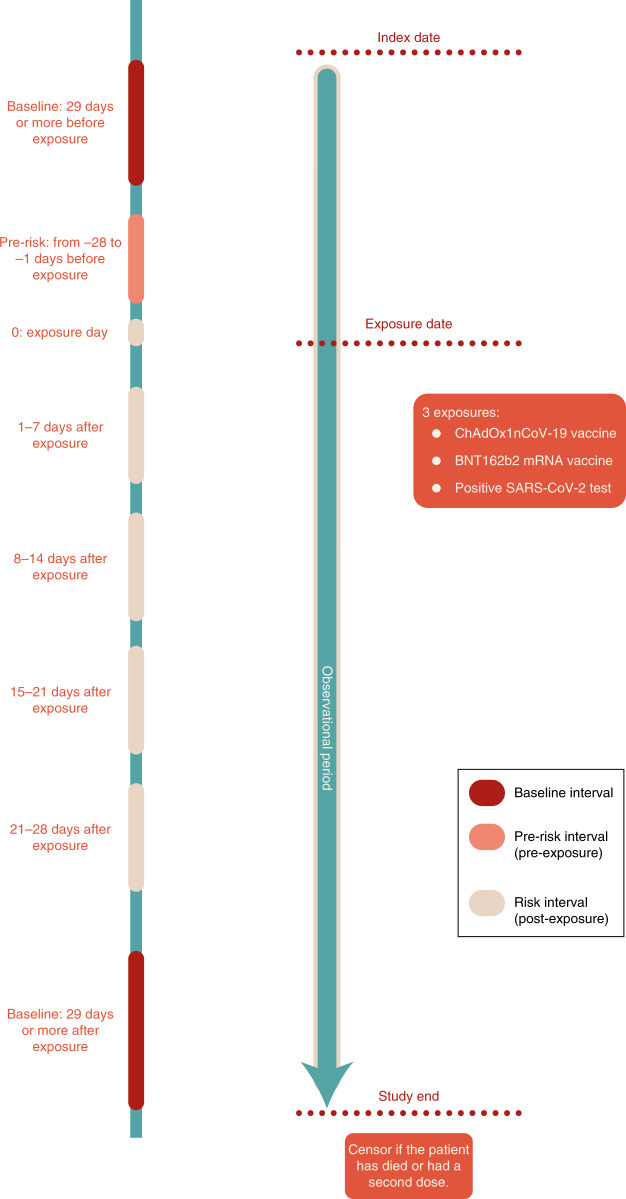
Schematic presentation of the SCCS study design. Each patient is followed from the index date to the study end date and is censored if death occurred or if they had a second dose of vaccine. Risk intervals (0, 1–7, 8–14, 15–21 and 22–28 days after exposure), the pre-risk interval (the 28 days prior to exposure) and the baseline periods (from study start to 29 days before exposure, and from 29 days after exposure to study end) are shown.

This study had several strengths. First, the United Kingdom was an ideal place to carry out this study given that both the ChAdOx1nCoV-19 and BNT162b2 vaccines have been rolled out at speed and scale. Second, this was a population-based study of prospectively recorded medical data and it avoided the recall and selection biases linked to case reports. Third, the large sample size provided sufficient power to investigate these rare neurological outcomes that could not be assessed through clinical trials. Fourth, the SCCS study design (Fig. 2) removes potential confounding from fixed characteristics, and the breakdown of the study period into weekly blocks accounted for temporal confounding. Last, the key finding that the ChAdOx1nCoV-19 vaccine was associated with an increased risk of subsequent Guillain–Barré syndrome was independently replicated using Scottish national data.

There were several limitations to this study. We examined the risks associated only with the first vaccine dose because the data on outcomes following second doses were limited at the time of this study. Furthermore, it was not possible to distinguish between Guillain–Barré syndrome variants including Miller Fisher syndrome in view of the coding used in the Hospital Episode Statistics database. Also, given that we investigated only hospital admissions and mortality, a proportion of patients with milder neurological disease may not have been included and the overall burden of neurological adverse events from vaccination and infection could be underestimated. Some hospital admissions for myasthenic disorders could represent exacerbations of previously well-controlled myasthenic illness given that we excluded only those with a prior hospital contact for these neurological conditions in the preceding 2 years. The Scottish replication cohort was smaller than the study cohort, which meant that estimates were less precise and we did not have sufficient statistical power to replicate findings with smaller effect sizes.

In summary, this population-based study identifies and quantifies several rare neurological adverse events that are specific to the ChAdOx1nCoV-19 and BNT162b2 vaccines, as well as SARS-CoV-2 infection. We believe that these findings are likely to be of relevance to other countries using these vaccines and it would be useful to replicate these results in similarly large datasets internationally. Clinicians should be aware of these rare complications, and the findings from this study will be paramount to policy makers in risk–benefit evaluations and health-care resource allocation. Importantly, the risks of adverse neurological events following SARS-CoV-2 infection are much greater than those associated with vaccinations, highlighting the benefits of ongoing vaccination programs.

## Methods

### Ethics approval

National Health Service Research Ethics Committee (NHS REC) approval was obtained from East Midlands-Derby Research Ethics Committee [reference 04/03/2021].

### Data

We used the NIMS Database of COVID-19 vaccination to identify vaccine exposure. This includes vaccine type, date and doses for all people vaccinated in England. National SARS-CoV-2 infection data were obtained from Public Health England (PHE).

This study used International Classification of Diseases 10 (ICD-10) codes to define neurological outcomes of interest (Supplementary Table 8). The diagnoses were extracted from Hospital Episode Statistics (HES), which is a database containing details of all admissions to National Health System (NHS) hospitals in England

**Extended Data Fig. 3:**
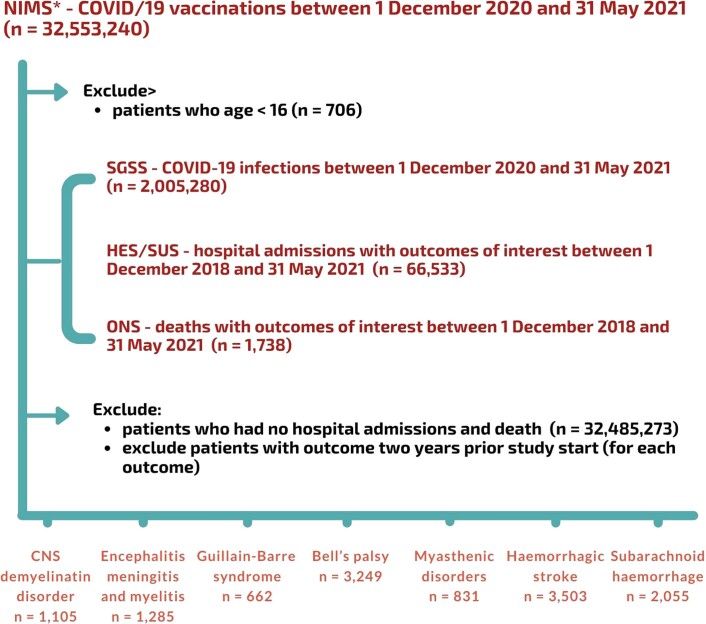
Data flow chart. Flowchart of data used in this study showing how data from National Immunisation Management System (NIMS), Second Generation Surveillance System (SGSS), Hospital Episode Statistics (HES) and Office of National Statistic (ONS) were used to obtain information about vaccinations, SARS-CoV-2 test results, and hospital admissions for neurological outcomes and deaths, respectively.

We linked NIMS, at the individual level, to national data for mortality (Office for National Statistics, ONS), hospital admissions (HES) and SARS-CoV-2 infection data (PHE). This information is now summarized in Extended Data Fig. 3.

### Study design

The SCCS design was originally developed to examine vaccine safety^14,15^. The method includes only individuals who have had the outcome of interest (cases) and compares the incidence rate (in each case) of the outcome of interest during a time-limited exposed period (for example, immediately after vaccination) with rates during unexposed periods (that is, baseline). A relative incidence greater than 1 indicates an increased risk after vaccination or a positive SARS-CoV-2 test. Given that the analyses are conditional on each case, any fixed characteristics during the study period, such as sex, ethnicity or chronic conditions, are inherently controlled for. Any time-varying factors, such as seasonal variation, need to be adjusted for in the analyses.

A flowchart showing how each dataset used for the SCCS analysis of each outcome was created is presented in Extended Data Fig. 3.

### Study period and population

We examined the associations between ChAdOx1nCoV-19 or BNT162b2 vaccines and neurological complications during the ongoing COVID-19 vaccination program in England (commenced on 8 December 2020). We also investigated the association between a SARS-CoV-2-positive test and the neurological diseases of interest in these vaccinated individuals. Separate analyses were carried out for each neurological outcome of interest. People were considered eligible for inclusion in each study cohort if they had received at least one vaccine dose, were at least 16 years old, and were admitted to hospital or died from the outcome of interest between 1 December 2020 and 31 May 2021 (last data update). Patients were followed up from the start (1 December 2020) to the earliest of either the end of the study period (31 May 2021), the date of second dose or when they died. Only the first outcome event during the study period was included. Patients with a hospital admission for the same outcome in the 2 years prior to the start of the study period were excluded. We excluded patients who received the mRNA-1273 (Moderna) vaccine given that the number of these patients was too small for meaningful analysis.

### Outcomes

The neurological outcomes in this study were selected a priori based on a literature search for any reports of emerging neurological complications from SARS-CoV-2 infection and vaccination. These included acute CNS demyelinating events, encephalitis meningitis and myelitis (excluding encephalopathy), Guillain–Barré syndrome (encapsulating all Guillain–Barré syndrome variants including Miller Fisher syndrome), Bell’s palsy, myasthenic disorders, hemorrhagic stroke and subarachnoid hemorrhage. Other outcomes, such as peripheral neuropathies, were excluded because these conditions were unlikely to result in hospital admission (particularly within the 28 day risk period in this study). We used the ICD-10 codes to define each outcome, as listed in Supplementary Table 8. The outcomes were identified as the first hospital admission due to the event of interest or as a cause of death recorded on the death certificate with the event.

### Exposures

The exposure variables were a first dose of the ChAdOx1nCoV-19 or BNT162b2 vaccines and first infection with SARS-CoV-2, defined as a positive COVID-19 polymerase chain reaction (PCR) test during the study period (either before or after vaccination). For all outcomes we defined the exposure risk intervals as the following pre-specified time periods: 0, 1–7, 8–14, 15–21 and 22–28 days after each exposure date, under the assumption that the adverse events under consideration are unlikely to be related to exposure beyond 28 days after the exposure. We assumed that patients who experienced an outcome before vaccination would probably delay vaccination until symptoms had improved. Therefore we included a pre-risk period in the analyses, defined as the 1–28 days immediately before the vaccination or SARS-CoV-2 test. The baseline period was then defined as extending from 1 December 2020 to 29 days before the exposure date, and from 29 days after the exposure date to 31 May 2021 or the censored date, if earlier. Hospital admissions for the events of interest can trigger COVID-19 testing. Such events may well be caused by SARS-CoV-2 infection, but the reverse causality involved in their detection induces bias. To reduce the bias, which could overestimate or underestimate the effect of infection, we allocated day 0 to a risk period on its own.

### Seasonality and COVID-19 pandemic period

**Extended Data Fig. 4:**
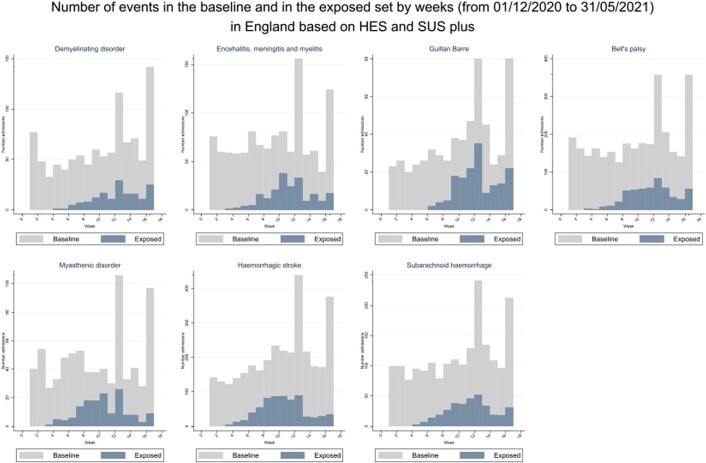
Number of hospital admissions or deaths for each outcome in the baseline and in the exposed set by week. Number of hospital admissions or deaths for each outcome in the baseline and in the exposed set by week from 1 December 2020 to 31 May 2021 (n = 32,553,534).

Some outcomes of interest have seasonal variation: for example, Guillain–Barré syndrome is more common in the winter months^21^. Moreover, hospital admissions were probably influenced by the pressure on the health systems due to COVID-19, which was not uniform during the pandemic period. To allow for these underlying seasonal effects, we split the observational study period into weeks and adjusted for week as a factor variable in the statistical models. In doing so, we estimated the relative incidence in exposure periods up to 28 days after vaccination compared with the unexposed periods, adjusted for underlying seasonality, which accounted for changes in hospital admission rates caused by the pandemic. If an exposure period is long in relation to the observation period, it might be impossible to distinguish between seasonal variation and vaccine effects. However, given that the exposure period was relatively short (28 days), this was unlikely to happen given that after 28 days a vaccinated patient returns to the baseline period, ensuring that in each interval both exposed and unexposed cases appear (Extended Data Fig. 4).

### Statistical analysis

We described the characteristics of each cohort (vaccinated patients with the outcomes of interest) in terms of age and sex. The SCCS models were fitted using a conditional Poisson regression model with an offset for the length of the risk period. IRRs, the rates of hospital admission or death due to each outcome of interest in risk periods relative to baseline periods, and their 95% CI were estimated using the SCCS model adjusted for week number as time-varying covariates. Exposure terms for both vaccines and for infection with SARS-CoV-2 were included in the same model.

We conducted sensitivity analyses to assess the robustness of results to assumptions such as that the occurrence of an outcome event did not influence the probability of subsequent exposures, by (1) excluding those who died from the outcome and (2) restricting analysis to the post-vaccination period, without censoring at death due to the outcome; to assess potential reporting delays in the data by (3) restricting the study period until 1 May 2021; and to test the choice of the follow-up period by (4) restricting analysis to patients who received only one vaccine dose and (5) censoring at 12 weeks after vaccination or at the date of a second dose if earlier. To test our assumption that events after 28 days are not related to the vaccines, we did an additional analysis with a 35 day risk period after exposure.

To further validate the estimated IRR associated with a positive SARS-CoV-2 test, we also restricted the analyses to only patients who had a positive SARS-CoV-2 test before vaccination.

Stata version 17 was used for these analyses.

### Absolute measures of effect

Absolute risk differences cannot be obtained using the SCCS methodology. We supplemented our estimates of IRRs with measures of the effect of each exposure in absolute terms, using a method^22^ developed to estimate the number of exposures needed to produce one excess adverse outcome and the excess number of events per 10 million exposed for each outcome.

### Interactions with age and sex

We investigated whether the association between vaccine exposures and outcomes is sex or age dependent by including, in the analysis, interaction terms between these covariates and the exposures. We used the likelihood ratio test to assess the interaction terms. We present results in separate subgroups when an interaction was significant.

### Negative and positive control

We examined the association of exposures with celiac disease as a negative control outcome^23^, which is assumed not to be associated with exposure to vaccination or SARS-CoV-2 infection; and with anaphylaxis as a positive control outcome given that it could occur shortly after vaccination with either vaccine^24^.

### Assessing the SCCS assumptions

**Extended Data Fig. 5:**
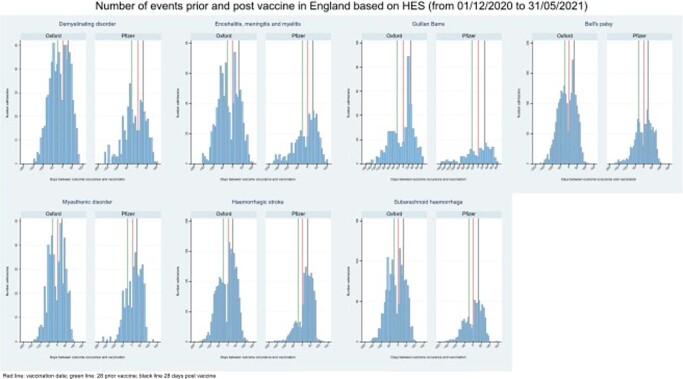
Number of hospital admissions or deaths for each outcome prior and post each vaccine. Number of hospital admissions or deaths for each outcome prior and post each vaccine from 1 December 2020 to 31 May 2021 (n = 32,553,534). Red line: vaccine date; green line: 28 days prior vaccine; black line: 28 days post vaccine.

**Extended Data Fig. 6:**
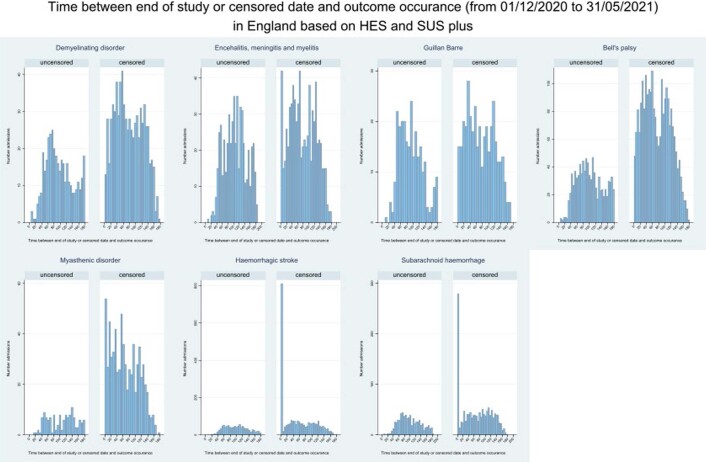
Time between end of study or censored date and date of hospital admission or deaths for each outcome. Time between end of study or censored date and date of hospital admission or deaths for each outcome from 1 December 2020 to 31 May 2021 (n = 32,553,534).

To further assess the assumptions of the SCCS and our modeling choices, we visually examined the data. We plotted a histogram of the number of occurrences of an event by time prior to or since vaccination for each outcome to assess the possibility that a hospital admission for that event might affect subsequent vaccination (Extended Data Fig. 5). We plotted a histogram of the time from the event to the actual end of observation in censored and uncensored cases to assess whether event-dependent observation periods may be a problem for the analysis (Extended Data Fig. 6).

#### Event-dependent exposures

Extended Data Fig. 5 shows the number of occurrences of an event by time before or since vaccination. We notice that there is a decrease in the 28 days immediately before vaccination, indicating that occurrence of an event might have reduced the likelihood of vaccination. This pattern is similar for most of the outcomes and for both vaccines. Therefore, we have added the pre-risk period of 28 days.

#### Event-dependent observation periods

Extended Data Fig. 6 shows the frequency of days from the event to the actual end of observation in censored and uncensored cases. A spike close to zero is apparent in the censored data histogram for hemorrhagic stroke and subarachnoid hemorrhage. This indicates the presence of event-dependent observation periods (censoring on death date due to outcome), which we tested further with sensitivity analyses 1 and 2. These additional analyses were in agreement with the main analysis, suggesting that there should be little concern for these outcomes.

### Replication using national Scottish data

To assess the robustness of the findings, we carried out an independent replication of the risks of neurological outcomes in vaccine recipients using a similar SCCS design. NHS Scotland provides comprehensive health care that is free at the point of care for all residents, with a base population that includes 5.4 million residents (~95% of the population) registered with a general medical practice in Scotland. We used deterministic linkage (based on the Community Health Index) to link vaccination information (extracted from general medical practice records and the Turas Vaccination Management Tool) and hospitalization records (from the Scottish Morbidity Records 01), mortality data (from National Records of Scotland) and SARS-Cov-2 infection data (from Electronic Communication of Surveillance in Scotland).

We classified exposures and outcomes in a similar way to the main analysis. An individual was defined as exposed to either the ChAdOx1nCoV-19 or BNT162b2 vaccine on the date that they received their first dose between 8 December 2020 and 31 May 2021. We defined outcomes of interest using the same ICD-10 codes as for the main analysis. We excluded patients who experienced the outcome in the 2 years prior to the study start date and excluded patients under 16 years of age. We fitted conditional Poisson regression models with an offset for the length of the risk period and adjusted for the weekly time period as a time-varying covariate. The control (baseline) period was the period from 1 December 2020 to 29 days before vaccination, allowing for a 28 day clearance (that is, pre-risk) period, and from 29 days after the exposure date to 31 May 2021 or the censored date, if earlier. We also stratified the post-vaccination follow-up period using the same time periods as the analysis in England when the number of events allowed. We also repeated the analysis by replacing vaccination with SARS-CoV-2 infection, which was defined as a positive PCR test within the study period. Given the smaller population size of Scotland, we focused on assessing the consistency of the point estimates of associations observed in the main analyses.

### Reporting Summary

Further information on research design is available in the Nature Research Reporting Summary linked to this article.

## Online content

Any methods, additional references, Nature Research reporting summaries, source data, extended data, supplementary information, acknowledgements, peer review information; details of author contributions and competing interests; and statements of data and code availability are available at 10.1038/s41591-021-01556-7.

## Supplementary information

Supplementary Information Supplementary Tables 1–8

Reporting Summary

## Data Availability

The data that support the findings of this study, that is, the National Immunisation (NIMS) Database of COVID-19, mortality (Office for National Statistics, ONS), hospital admissions (Hospital Episode Statistics, HES) and SARS-CoV-2 infection data (Public Health England, PHE) are not publicly available because they are based on de-identified national clinical records. Due to national and organizational data privacy regulations, individual-level data such as those used for this study cannot be shared openly. Approval to access PHE data needs to be provided by the PHE Office for Data Release. Approval to access NIMS, HES and ONS data requires permission from NHS Digital, who retain the copyright in that data.

## Extended data

are available for this paper at 10.1038/s41591-021-01556-7.

## Code availability

The code used for this study has been deposited in the Git repository of the research group, which is protected by privacy. Access to the code is available from the authors on request. Stata version 17 was used for these analyses.
