# Publisher Correction: Neurological complications after first dose of COVID-19 vaccines and SARS-CoV-2 infection

**DOI:** 10.1038/s41591-021-01644-8

**Published:** 2021-11-29

**Authors:** Martina Patone, Lahiru Handunnetthi, Defne Saatci, Jiafeng Pan, Srinivasa Vittal Katikireddi, Saif Razvi, David Hunt, Xue W. Mei, Sharon Dixon, Francesco Zaccardi, Kamlesh Khunti, Peter Watkinson, Carol A. C. Coupland, James Doidge, David A. Harrison, Rommel Ravanan, Aziz Sheikh, Chris Robertson, Julia Hippisley-Cox

**Affiliations:** 1 Nuffield Department of Primary Health Care Sciences, University of Oxford, Oxford, UK; 2 Nuffield Department of Clinical Neurosciences, University of Oxford, Oxford, UK; 3 Wellcome Centre for Human Genetics, University of Oxford, Oxford, UK; 4 Department of Mathematics and Statistics, University of Strathclyde, Glasgow, UK; 5 MRC/CSO Social and Public Health Sciences Unit, University of Glasgow, Glasgow, UK; 6 Institute of Neurological Sciences, Glasgow, UK; 7 Centre for Clinical Brain Sciences and UK Dementia Research Institute, University of Edinburgh, Edinburgh, UK; 8 Leicester Real World Evidence Unit, Leicester Diabetes Centre, University of Leicester, Leicester, UK; 9 NIHR Biomedical Research Centre, Oxford University Hospitals NHS Trust, Oxford, UK; 10 Division of Primary Care, School of Medicine, University of Nottingham, Nottingham, UK; 11 Intensive Care National Audit and Research Centre, London, UK; 12 London School of Hygiene and Tropical Medicine, London, UK; 13 NHS Blood and Transplant, Bristol, UK; 14 Usher Institute, University of Edinburgh, Edinburgh, UK; 15 Public Health Scotland, Glasgow, UK

**Keywords:** Neuroscience, Neurological disorders

Correction to: *Nature Medicine* 10.1038/s41591-021-01556-7, published online 25 October 2021.

In the version of this Article initially published online, there were errors in the author affiliations shown for authors Xue W. Mei, Sharon Dixon, Carol A. C. Coupland and Julia Hippisley-Cox. They are affiliated with the Nuffield Department of Primary Health Care Sciences, University of Oxford, Oxford, UK (affiliation 1), not the Nuffield Department of Clinical Neurosciences (affiliation 2). The changes have been made to the online version of the Article.

