# Supplementary material for: Neurological complications after first dose of COVID-19 vaccines and SARS-CoV-2 infection
Source: Nat Med. 2021 Oct 25;27(12):2144–53. doi: 10.1038/s41591-021-01556-7 (PMC8629105; doi:10.1038/s41591-021-01556-7)
Supplement: Supplementary file 1 — Supplementary Tables 1–8 [file 41591_2021_1556_MOESM1_ESM.pdf]

nature portfolio

https://doi.org/10.1038/s41591-021-01556-7

Supplementary information

Neurological complications after first dose of COVID-19 vaccines and SARS-CoV-2 infection

In the format provided by the authors and unedited

Supplementary table 1: Incidence rate ratios (IRR 95% CI) for Bell's palsy excluding patients who had a suspected concurrent diagnosis of cerebral infarction in pre-defined risk period (1-28 days) immediately before and after exposure to vaccination and before and after a Positive SARS-CoV-2 test result, adjusted for calendar time from 1 December 2020 until 31 May 2021 (cells with < 5 are suppressed).

		**ChAdOx1nCoV-19 vaccine**		**BNT162b2 mRNA vaccine**		**Positive SARS-CoV-2 test**	
	Time period	events	IRR (95% CI)	events	IRR (95% CI)	events	IRR (95% CI)
**Bell's palsy**	Baseline	1269	1.00	674	1.00	184	1.00
	-28 to -1 days	303	0.79 (0.70, 0.90)	160	0.78 (0.65, 0.94)	74	3.12 (2.29, 4.25)
	0 day	*	0.28 (0.11, 0.76)	*	0.39 (0.12, 1.20)	34	35.18 (23.70, 52.24)
	1-7 days	87	0.87 (0.70, 1.09)	51	0.92 (0.68, 1.23)	43	6.25 (4.35, 8.98)
	8-14 days	81	0.81 (0.64, 1.02)	53	0.93 (0.70, 1.24)	17	2.38 (1.42, 3.99)
	15-21 days	132	1.33 (1.10, 1.60)	61	1.04 (0.79, 1.37)	8	1.06 (0.51, 2.17)
	22-28 days	110	1.14 (0.93, 1.40)	70	1.22 (0.97, 1.57)	5	0.62 (0.25, 1.51)

Supplementary table 2: Incidence rate ratios (IRR 95% CI) of the combined occurrence of Guillain-Barre syndrome and Bell's palsy in pre-defined risk periods immediately before and after exposure to vaccination and before and after a positive SARS-CoV-2 test result, adjusted for calendar time from 1 December 2020 to 31 May 2021 (cells with < 5 are suppressed).

		**ChAdOx1nCoV-19 vaccine**		**BNT162b2 mRNA vaccine**		**Positive SARS-CoV-2 test**	
	**Time period**	**events**	**IRR (95% CI)**	**events**	**IRR (95% CI)**	**events**	**IRR (95% CI)**
**Guillain-Barre and Bell's palsy**	Baseline	12	1.00	34	1.00	31	1.00
	-28 to -1 days	*	0.76 (0.04, 16.09)	0	n/a	1	n/a
	0 day	*	n/a	0	n/a	0	n/a
	1-28 days	21	12.66 (2.70, 59.21)	0	n/a	2	n/a

**Guillain-Barre and Bell's palsy**	Baseline	12	1.00	34	1.00	31	1.00
	-28 to -1 days	*	0.70 (0.03, 14.93)	0	n/a	*	n/a
	0 day	*	n/a	0	n/a	0	n/a
	1-7 days	0	n/a	0	n/a	0	n/a
	8-14 days	0	n/a	0	n/a	0	n/a
	15-21 days	13	28.86 (5.45, 152.74)	0	n/a	*	n/a
	22-28 days	8	13.35 (2.48, 72.92)	0	n/a	*	n/a

Supplementary table 3a: Baseline demographic characteristics of people receiving either ChAdOx1nCoV-19 or BNT162b2 vaccines or testing positive to SARS-CoV-2 virus (in those vaccinated with either vaccine), in Scotland between 1 December 2020 until 31 May 2021. Figures are column % (counts).

	**ChAdOx1nCoV-19 vaccine**	**BNT162b2 mRNA vaccine**	**Positive SARS-CoV-2 test (amongst vaccinated)**
	Col % (counts)	*Col % (counts)*	*Col % (counts)*
Total number of people	1982678	1077626	117554
**Sex**			
Number of women	50.7 (1005836)	59.1 (637102)	58.6 (68941)
Number of men	49.3 (976842)	40.9 (440524)	41.4 (48613)
**Age**			
16-29 years	4.00 (79133)	10.9 (117779)	11.0 (12976)
30-39 years	7.5 (148417)	16.4 (177002)	14.2 (16709)
40-49 years	18.7 (371830)	17.3 (186315)	21.4 (25133)
50-59 years	29.0 (574910)	13.7 (148197)	26.3 (30939)
60-69 years	18.4 (364418)	22.0 (236708)	13.0 (15299)
70-79 years	13.1 (259069)	16.2 (175170)	7.0 (8187)
80-89 years	8.2 (163025)	2.6 (28396)	5.4 (6317)
90+ years	1.1 (21876)	0.7 (8059)	1.7 (1994)

Q risk			
0	49.9 (990306)	55.3 (596051)	51.7 (60802)
1	28.9 (572508)	27.5 (296364)	28.0 (32881)
2	12.4 (246298)	10.3 (111229)	10.9 (12833)
3	5.1 (101885)	4.0 (43507)	4.7 (5590)
4	2.2 (43467)	1.7 (18351)	2.5 (2921)
5+	1.4 (28214)	1.1 (12124)	2.1 (2527)
Prior demyelinating disorder	<0.1 (253)	<0.1 (93)	<0.1 (16)
Prior encephalitis meningitis and myelitis	<0.1 (294)	<0.1 (147)	<0.1 (27)
Prior Guillain-Barre	<0.1 (136)	<0.1 (55)	<0.1 (16)
Prior Bell's palsy	<0.1 (530)	0.1 (245)	0.1 (42)
Prior myasthenic disorder	<0.1 (352)	<0.1 (111)	<0.1 (26)
Prior haemorrhagic stroke	<0.1 (820)	<0.1 (380)	0.1 (88)
Prior subarachnoid haemorrhage	<0.1 (531)	<0.1 (248)	<0.1 (42)

Supplementary table 3b: Incidence rate ratios (IRR 95% CI) for individual outcomes in pre-defined risk period (1-28 days) immediately before and after exposure to vaccination and before and after a Positive SARS-CoV-2 test result, adjusted for calendar time from 1 December 2020 until 31 May 2021 using Scottish national data (cells with < 5 are suppressed).

		Either vaccine		ChAdOx1nCoV-19 vaccine		BNT162b2 mRNA vaccine		Positive SARS-CoV-2 test	
	**Time period**	**events**	**IRR (95% CI)**	**events**	**IRR (95% CI)**	**events**	**IRR (95% CI)**	**events**	**IRR (95% CI)**
**CNS Demyelinating disorder**	Baseline	32	1.00	21	1.00	11	1.00	*	1.00
	-28 to -1 days	15	2.12 (1.02, 4.42)	14	2.19 (0.91, 5.28)	*	0.62 (0.06, 6.89)	*	4.67 (0.29, 74.61)
	0 day	*	n/a	*	n/a	*	n/a	*	n/a
	1-28 days	6	0.9 (0.34, 2.43)	6	1.16 (0.38, 3.52)	*	n/a	*	4.5 (0.28, 71.94)

**Encephalitis meningitis and myelitis**	Baseline	71	1.00	52	1.00	19	1.00	6	1.00
	-28 to -1 days	6	0.25 (0.10, 0.59)	6	0.36 (0.14, 0.87)	*	n/a	*	n/a
	0 day	*	n/a	*	n/a	*	n/a	*	n/a
	1-28 days	14	0.6 (0.32, 1.14)	9	0.61 (0.27, 1.35)	5	0.75 (0.22, 2.47)	*	0.63 (0.07, 5.35)
**Guillain-Barre**	Baseline	21	1.00	19	1.00	*	1.00	*	1.00
	-28 to -1 days	*	n/a	*	n/a	*	n/a	*	n/a
	0 day	*	n/a	*	n/a	*	n/a	*	n/a
	1-28 days	19	2.32 (1.08, 5.02)	17	2.31 (1.02, 5.24)	*	1.00 (0.02, 41.99)	*	n/a
**Bell's palsy**	Baseline	98	1.00	74	1.00	24	1.00	*	1.00
	-28 to -1 days	19	0.66 (0.39, 1.12)	14	0.63 (0.34, 1.17)	5	1.06 (0.35, 3.18)	*	3.07 (0.27, 34.37)
	0 day	*	0.91 (0.12, 6.57)	*	1.15 (0.16, 8.43)	*	n/a	*	65.77 (5.96, 725.58)
	1-28 days	35	1.13 (0.73, 1.75)	26	1.13 (0.68, 1.87)	9	1.17 (0.44, 3.09)	*	4.7 (0.66, 33.36)
**Myasthenic disorder**	Baseline	40	1.00	28	1.00	12	1.00	*	1.00
	-28 to -1 days	7	0.38 (0.14, 1.04)	*	0.24 (0.06, 0.91)	*	n/a	*	n/a
	0 day	*	n/a	*	n/a	*	n/a	*	n/a
	1-28 days	11	0.46 (0.19, 1.13)	10	0.52 (0.18, 1.52)	*	n/a	*	n/a
**Haemorrhagic stroke**	Baseline	258	1.00	206	1.00	52	1.00	16	1.00
	-28 to -1 days	27	0.23 (0.15, 0.35)	23	0.24 (0.15, 0.39)	*	0.16 (0.05, 0.48)	7	1.6 (0.64, 4.03)
	0 day	*	0.18 (0.03, 1.32)	*	0.24 (0.03, 1.74)	*	n/a	*	5.7 (0.75, 43.45)
	1-28 days	98	0.64 (0.48, 0.85)	73	0.63 (0.46, 0.87)	25	0.65 (0.35, 1.2)	*	0.7 (0.20, 2.41)
**Subarachnoid haemorrhage**	Baseline	159	1.00	118	1.00	41	1.00	12	1.00
	-28 to -1 days	17	0.22 (0.13, 0.37)	16	0.3 (0.17, 0.53)	*	0.05 (0.01, 0.4)	*	1.56 (0.49, 4.94)
	0 day	*	n/a	*	n/a	*	n/a	*	29.84 (8.25, 107.93)

	1-28 days	52	0.6 (0.42, 0.86)	39	0.78 (0.51, 1.19)	13	0.3 (0.13, 0.7)	*	n/a

Supplementary table 3c: Incidence rate ratios (IRR 95% CI) for Guillain-Barre syndrome in pre-defined risk periods immediately before and after exposure to vaccination and before and after a Positive SARS-CoV-2 test result, adjusted for calendar time from 1 December 2020 until 31 May 2021 using Scottish national data (cells with < 5 are suppressed).

		Either vaccine		ChAdOx1nCoV-19 vaccine		BNT162b2 mRNA vaccine		Positive SARS-CoV-2 test	
	**Time period**	**events**	**IRR (95% CI)**	**events**	**IRR (95% CI)**	**events**	**IRR (95% CI)**	**events**	**IRR (95% CI)**
**Guillain-Barre**	Baseline	21	1.00	19	1.00	*	1.00	*	1.00
	-28 to -1 days	*	n/a	*	n/a	*	n/a	*	n/a
	0 day	*	n/a	*	n/a	*	n/a	*	n/a
	1-7 days	*	n/a	*	n/a	*	n/a	*	n/a
	8-14 days	*	1.91 (0.58, 6.27)	*	2.15 (0.63, 7.31)	*	n/a	*	n/a
	15-21 days	10	5.35 (2.09, 13.70)	8	4.79 (1.72, 13.38)	*	n/a	*	n/a
	22-28 days	5	3.40 (1.09, 10.65)	5	3.60 (1.11, 11.65)	*	n/a	*	n/a

Supplementary table 4a: Likelihood ratio test statistic and p-value to test interaction between vaccine type and age group or sex. Interaction term was tested only when the main exposure (vaccine) was found to be significant (cells with < 5 are suppressed).

	**Likelihood ratio test (df = 6)**	
	with ChAdOx1nCoV-19 vaccine	with BNT162b2 mRNA vaccine
**Interaction with age group (≤50, >50)**		
CNS Demyelinating disorder	-	-
Encephalitis meningitis and myelitis	7.47 (0.279)	-
Guillain-Barre	1.88 (0.930)	-
Bell's palsy	3.37 (0.761)	-
Myasthenic disorder	15.57 (0.016)	-
Haemorrhagic stroke	-	8.24 (0.221)

Subarachnoid haemorrhage	-	-
**Interaction with sex**		-
CNS Demyelinating disorder	-	-
Encephalitis meningitis and myelitis	5.30 (0.506)	-
Guillain-Barre	4.43 (0.618)	-
Bell's palsy	7.02 (0.319)	-
Myasthenic disorder	5.57 (0.473)	-
Haemorrhagic stroke	-	17.72 (0.007)
Subarachnoid haemorrhage	-	-

Supplementary table 4b: Incidence rate ratios (IRR 95% CI) by age groups or sex for the outcomes in pre-defined risk periods immediately before and after exposure to vaccination and before and after a positive SARS-CoV-2 test result, adjusted for calendar time from 1 December 2020 to 31 May 2021 (cells with < 5 are suppressed).

		**ChAdOx1nCoV-19 vaccine**		**BNT162b2 mRNA vaccine**		**Positive SARS-CoV-2 test**	
	**Time period**	**events**	**IRR (95% CI)**	**events**	**IRR (95% CI)**	**events**	**IRR (95% CI)**
**Interaction with age group**							
**Myasthenic disorder**							
**Age ≤ 50**	baseline	44	1.00	29	1.00	8	1.00
	-28 to -1 days	12	1.09 (0.54, 2.17)	9	1.39 (0.63, 3.09)	*	0.77 (0.09, 6.50)
	0 day	0	n/a	0	n/a	*	17.43 (2.04, 148.80)
	1-7 days	0	n/a	*	1.81 (0.51, 6.45)	5	11.20 (3.29, 38.07)
	8-14 days	*	n/a	*	1.83 (0.52, 6.47)	0	n/a
	15-21 days	8	2.44 (1.07, 5.55)	*	1.62 (0.36, 7.23)	*	2.09 (0.25, 17.44)
	22-28 days	*	0.84 (0.25, 2.81)	*	1.26 (0.29, 5.56)	*	1.97 (0.24, 16.13)
**Age > 50**	baseline	267	1.00	184	1.00	24	1.00
	-28 to -1 days	58	0.65 (0.47, 0.90)	35	0.49 (0.33, 0.72)	25	4.75 (2.62, 8.64)
	0 day	0	n/a	*	0.98 (0.31, 3.12)	17	70.94 (36.93, 136.28)

	1-7 days	17	0.76 (0.45, 1.29)	15	0.71 (0.41, 1.24)	24	13.90 (7.66, 25.24)
	8-14 days	26	1.28 (0.82, 1.97)	21	0.99 (0.61, 1.60)	9	4.92 (2.24, 10.83)
	15-21 days	25	1.34 (0.86, 2.07)	20	0.94 (0.57, 1.53)	*	2.16 (0.74, 6.29)
	22-28 days	20	1.00 (0.62, 1.62)	25	1.40 (0.90, 2.16)	5	2.97 (1.12, 7.87)
**Interaction with sex**							
**Haemorrhagic stroke**							
**Female**	baseline	695	1.00	401	1.00	95	1.00
	-28 to -1 days	109	0.49 (0.39, 0.60)	30	0.27 (0.18, 0.39)	80	4.69 (3.35, 6.57)
	0 day	6	0.69 (0.31, 1.55)	*	0.42 (0.10, 1.69)	9	11.12 (5.52, 22.40)
	1-7 days	63	1.03 (0.78, 1.34)	50	1.44 (1.05, 1.96)	11	1.90 (1.00, 3.61)
	8-14 days	89	1.44 (1.14, 1.82)	44	1.23 (0.89, 1.71)	7	1.16 (0.53, 2.53)
	15-21 days	70	1.14 (0.88, 1.47)	67	1.84 (1.40, 2.42)	5	0.84 (0.34, 2.07)
	22-28 days	66	1.10 (0.85, 1.44)	49	1.30 (0.96, 1.77)	*	0.52 (0.16, 1.64)
**Male**	baseline	760	1.00	409	1.00	78	1.00
	-28 to -1 days	123	0.46 (0.38, 0.57)	56	0.46 (0.34, 0.61)	83	4.99 (3.53, 7.04)
	0 day	*	0.10 (0.01, 0.70)	*	0.76 (0.28, 2.04)	11	14.41 (7.54, 27.52)
	1-7 days	68	0.95 (0.73, 1.23)	43	1.13 (0.82, 1.57)	12	2.24 (1.20, 4.18)
	8-14 days	52	0.73 (0.55, 0.98)	45	1.13 (0.82, 1.56)	7	1.26 (0.58, 2.76)
	15-21 days	58	0.84 (0.63, 1.10)	39	0.98 (0.70, 1.38)	5	0.87 (0.35, 2.17)
	22-28 days	60	0.91 (0.70, 1.20)	42	1.03 (0.74, 1.42)	*	0.52 (0.16, 1.66)

Supplementary table 5: Measures of effect of vaccinations and SARS-CoV-2 infections presented in absolute terms, number needed to expose for one excess event; excess events due to exposure; excess events per 10 million exposed. Only associations significant at the 0.01 level were reported over the 1-28 days post exposure. When IRR were not significant over the 1-28 days post-vaccine, absolute measures were not given.

	IRR (95% CI) 1-28 days*	Number needed to expose to cause one excess event (95% CI)	Excess events per 10,000,000 exposed (95% CI)
**CNS Demyelinating disorder**			
with ChAdOx1nCoV-19 vaccine	0.97 (0.78, 1.22)	-	-
with BNT162b2 mRNA vaccine	1.02 (0.75, 1.40)	-	-
with SARS-CoV-2 infection	1.67 (0.93, 3.00)	-	-
**Encephalitis meningitis and myelitis**			
with ChAdOx1nCoV-19 vaccine	1.07 (0.87, 1.31)	-	-
with BNT162b2 mRNA vaccine	1.14 (0.86, 1.51)	-	-
with SARS-CoV-2 infection	2.70 (1.78, 4.11)	81166 (44168, 76269)	123 (131, 226)
**Guillain-Barre**			
with ChAdOx1nCoV-19 vaccine	2.04 (1.60, 2.60)	261766 (216855, 355865)	38 (28, 46)
with BNT162b2 mRNA vaccine	0.86 (0.54, 1.36)	-	-
with SARS-CoV-2 infection	5.25 (3.00, 9.18)	68809 (62512, 83526)	145 (120, 160)
**Bell's palsy**			
with ChAdOx1nCoV-19 vaccine	1.07 (0.94, 1.21)	-	-
with BNT162b2 mRNA vaccine	1.06 (0.90, 1.26)	-	-
with SARS-CoV-2 infection	1.34 (0.91, 1.97)	-	-
**Myasthenic disorder**			
with ChAdOx1nCoV-19 vaccine	1.23 (0.94, 1.62)	-	-
with BNT162b2 mRNA vaccine	1.18 (0.88, 1.59)	-	-
with SARS-CoV-2 infection	3.01 (1.70, 5.36)	61284 (50310, 99387)	163 (101, 199)
**Haemorrhagic stroke**			
with ChAdOx1nCoV-19 vaccine	1.02 (0.90, 1.15)	-	-
with BNT162b2 mRNA vaccine	1.24 (1.07, 1.43)	165426 (106478, 489416)	60 (20, 94)
with SARS-CoV-2 infection	0.85 (0.57, 1.26)	-	-
**Subarachnoid haemorrhage**			
with ChAdOx1nCoV-19 vaccine	1.01 (0.86, 1.18)	-	-
with BNT162b2 mRNA vaccine	1.05 (0.84, 1.30)	-	-
with SARS-CoV-2 infection	1.51 (0.96, 2.36)	-	-

Supplementary Table 6: Incidence rate ratios (IRR 95% CI) for celiac disease (negative control) and anaphylaxis (positive control) in pre-defined risk periods immediately before and after exposure to vaccination and before and after a positive SARS-CoV-2 result, adjusted for calendar time from 1 December 2020 to 31 Maggio 2021 (cells with < 5 are suppressed).

		Celiac disease (negative control)		Anaphylaxis (positive control)	
		**events**	**IRR (95% CI)**	**events**	**IRR (95% CI)**
**Group**	**Time period**				
**ChAdOx1nCoV-19 vaccine**	baseline	2403	1.00	438	1.00
	-28 to -1 days	715	0.95 (0.87, 1.04)	111	0.82 (0.66, 1.02)
	0 day	*	0.07 (0.02, 0.29)	47	9.68 (7.08, 13.24)
	1-7 days	164	0.84 (0.72, 0.99)	52	1.55 (1.15, 2.09)
	8-14 days	196	1.01 (0.87, 1.17)	25	0.74 (0.49, 1.11)
	15-21 days	184	0.97 (0.83, 1.13)	36	1.05 (0.74, 1.49)
	22-28 days	183	0.99 (0.85, 1.16)	26	0.78 (0.52, 1.18)
**BNT162b2 mRNA vaccine**	baseline	1610	1.00	167	1.00
	-28 to -1 days	437	0.88 (0.79, 0.98)	31	0.73 (0.49, 1.08)
	0 day	5	0.27 (0.11, 0.65)	29	19.31 (12.77, 29.19)
	1-7 days	98	0.75 (0.61, 0.92)	16	1.50 (0.89, 2.55)
	8-14 days	121	0.90 (0.75, 1.09)	22	1.99 (1.25, 3.17)
	15-21 days	122	0.89 (0.73, 1.07)	12	1.10 (0.60, 2.00)
	22-28 days	116	0.86 (0.71, 1.05)	11	0.99 (0.53, 1.84)
**SARS-CoV-2**	baseline	207	1.00	59	1.00
	-28 to -1 days	89	2.08 (1.60, 2.70)	16	1.49 (0.83, 2.67)
	0 day	42	25.62 (18.22, 36.03)	11	25.81 (13.25, 50.30)
	1-7 days	69	5.91 (4.45, 7.84)	8	2.68 (1.26, 5.70)
	8-14 days	53	4.33 (3.18, 5.90)	*	0.67 (0.16, 2.76)

	15-21 days	18	1.38 (0.85, 2.25)	*	0.95 (0.30, 3.07)
	22-28 days	19	1.35 (0.84, 2.16)	6	1.77 (0.76, 4.13)

Supplementary table 7a: Incidence rate ratios (IRR 95% CI) for single outcomes in pre-defined risk periods immediately before and after exposure to vaccination, adjusted for calendar time from 1 December 2020 to 31 May 2021. Comparisons between different sensitivity analyses (cells with < 5 are suppressed).

		Main		Sensitivity 1*		Sensitivity 2*		Sensitivity 3*		Sensitivity 4*		Sensitivity 5*	
		events	IRR (95% CI)	events	IRR (95% CI)	events	IRR (95% CI)	events	IRR (95% CI)	events	IRR (95% CI)	events	IRR (95% CI)
**CNS Demyelinating disorder°**													
**ChAdOx1nCoV-19 vaccine**	Baseline	473	1.00	473	1.00			464	1.00	174	1.00	473	1.00
	-28 to -1 days	117	0.83 (0.67, 1.03)	117	0.83 (0.67, 1.03)			117	0.83 (0.67, 1.03)	43	0.71 (0.50, 1.03)	117	0.82 (0.66, 1.02)
	0 day	0	n/a	0	n/a			0	n/a	0	n/a	0	n/a
	1-7 days	38	1.03 (0.73, 1.44)	38	1.02 (0.73, 1.44)			37	1.01 (0.71, 1.42)	16	1.11 (0.64, 1.91)	38	1.02 (0.73, 1.44)
	8-14 days	35	0.95 (0.67, 1.36)	35	0.95 (0.67, 1.35)			35	0.96 (0.67, 1.37)	11	0.81 (0.43, 1.54)	35	0.95 (0.67, 1.35)
	15-21 days	38	1.04 (0.74, 1.46)	38	1.04 (0.74, 1.46)			38	1.04 (0.74, 1.46)	16	1.25 (0.73, 2.17)	38	1.03 (0.73, 1.45)
	22-28 days	33	0.93 (0.65, 1.33)	33	0.93 (0.64, 1.33)			32	0.90 (0.62, 1.30)	13	1.13 (0.62, 2.05)	33	0.92 (0.64, 1.32)
**BNT162b2 mRNA vaccine**	Baseline	244	1.00	242	1.00			240	1.00	81	1.00	242	1.00
	-28 to -1 days	58	0.92 (0.68, 1.24)	58	0.93 (0.69, 1.25)			58	0.93 (0.69, 1.26)	6	0.47 (0.20, 1.10)	58	0.91 (0.67, 1.23)
	0 day	*	0.43 (0.06, 3.05)	*	0.43 (0.06, 3.09)			*	0.43 (0.06, 3.11)	*	2.61 (0.36, 19.13)	*	0.42 (0.06, 3.02)
	1-7 days	15	0.91 (0.53, 1.55)	15	0.92 (0.54, 1.57)			15	0.93 (0.54, 1.58)	*	0.37 (0.05, 2.72)	15	0.90 (0.53, 1.53)
	8-14 days	16	0.94 (0.56, 1.58)	15	0.89 (0.52, 1.51)			16	0.95 (0.57, 1.60)	5	1.89 (0.73, 4.89)	16	0.93 (0.55, 1.56)
	15-21 days	16	0.92	16	0.93			16	0.93	*	1.53	16	0.91

			(0.55, 1.55)		(0.55, 1.56)				(0.56, 1.57)		(0.54, 4.38)		(0.54, 1.53)
	22-28 days	21	1.19 (0.75, 1.89)	21	1.20 (0.76, 1.90)			21	1.20 (0.76, 1.90)	*	1.14 (0.35, 3.76)	21	1.18 (0.74, 1.86)
**Encephalitis meningitis and myelitis**													
**ChAdOx1nCoV-19 vaccine**	Baseline	602	1.00	586	1.00	171	1.00	594	1.00	291	1.00	599	1.00
	-28 to -1 days	78	0.42 (0.33, 0.54)	78	0.43 (0.34, 0.56)			78	0.42 (0.33, 0.54)	25	0.31 (0.20, 0.47)	78	0.42 (0.33, 0.54)
	0 day	*	0.46 (0.15, 1.43)	*	0.48 (0.15, 1.50)	*	0.34 (0.10, 1.11)	*	0.46 (0.15, 1.44)	*	0.36 (0.05, 2.59)	*	0.46 (0.15, 1.43)
	1-7 days	52	1.15 (0.85, 1.54)	47	1.09 (0.80, 1.49)	52	0.86 (0.55, 1.35)	52	1.16 (0.86, 1.56)	28	1.48 (0.98, 2.23)	52	1.14 (0.85, 1.54)
	8-14 days	58	1.32 (0.99, 1.76)	57	1.38 (1.03, 1.84)	58	1.06 (0.71, 1.58)	58	1.34 (1.00, 1.78)	34	1.84 (1.26, 2.70)	58	1.32 (0.99, 1.75)
	15-21 days	37	0.89 (0.63, 1.25)	37	0.94 (0.66, 1.32)	37	0.76 (0.50, 1.15)	37	0.90 (0.64, 1.27)	14	0.81 (0.47, 1.41)	37	0.88 (0.63, 1.25)
	22-28 days	38	0.96 (0.68, 1.35)	36	0.96 (0.68, 1.37)	38	0.86 (0.58, 1.26)	38	0.97 (0.69, 1.37)	22	1.41 (0.90, 2.23)	38	0.95 (0.68, 1.34)
**BNT162b2 mRNA vaccine**	Baseline	275	1.00	268	1.00	135	1.00	273	1.00	120	1.00	271	1.00
	-28 to -1 days	45	0.59 (0.42, 0.82)	45	0.60 (0.43, 0.83)			45	0.59 (0.43, 0.83)	8	0.45 (0.22, 0.95)	45	0.58 (0.41, 0.80)
	0 day	0	n/a	0	n/a	0	n/a	0	n/a	0	n/a	0	n/a
	1-7 days	22	1.02 (0.65, 1.61)	20	0.95 (0.59, 1.52)	22	0.64 (0.35, 1.18)	22	1.03 (0.66, 1.62)	9	2.01 (0.98, 4.13)	22	1.00 (0.63, 1.56)
	8-14 days	27	1.17 (0.77, 1.77)	25	1.10 (0.72, 1.69)	27	0.81 (0.48, 1.38)	27	1.18 (0.78, 1.78)	8	1.65 (0.77, 3.51)	27	1.14 (0.75, 1.73)
	15-21 days	21	0.95 (0.60, 1.50)	19	0.88 (0.55, 1.43)	21	0.69 (0.40, 1.17)	21	0.96 (0.60, 1.51)	11	2.32 (1.20, 4.51)	21	0.93 (0.59, 1.47)
	22-28 days	27	1.28 (0.85, 1.93)	25	1.22 (0.80, 1.87)	27	0.96 (0.61, 1.53)	27	1.28 (0.85, 1.93)	9	1.92 (0.93, 3.93)	27	1.25 (0.83, 1.88)
**Guillain-Barre°**													
**ChAdOx1nCoV-19 vaccine**	Baseline	264	1.00	260	1.00			261	1.00	101	1.00	264	1.00
	-28 to -1 days	41	0.44 (0.31, 0.63)	41	0.45 (0.32, 0.64)			41	0.44 (0.31, 0.63)	10	0.29 (0.15, 0.57)	41	0.45 (0.31, 0.63)
	0 day	0	n/a	0	n/a			0	n/a	0	n/a	0	n/a

	1-7 days	17	0.74 (0.44, 1.23)	17	0.76 (0.46, 1.26)			17	0.73 (0.44, 1.22)	7	0.84 (0.38, 1.84)	17	0.74 (0.45, 1.23)
	8-14 days	23	1.02 (0.65, 1.59)	22	1.01 (0.64, 1.59)			23	1.01 (0.65, 1.58)	14	1.72 (0.95, 3.11)	23	1.02 (0.65, 1.60)
	15-21 days	65	2.90 (2.15, 3.92)	62	2.86 (2.11, 3.89)			64	2.84 (2.10, 3.84)	51	6.55 (4.43, 9.69)	65	2.90 (2.15, 3.91)
	22-28 days	48	2.21 (1.59, 3.09)	48	2.29 (1.64, 3.20)			48	2.20 (1.58, 3.07)	30	4.15 (2.63, 6.56)	48	2.20 (1.58, 3.07)
**BNT162b2 mRNA vaccine**	Baseline	109	1.00	107	1.00			108	1.00	41	1.00	108	1.00
	-28 to -1 days	21	0.67 (0.41, 1.10)	21	0.68 (0.41, 1.11)			21	0.68 (0.42, 1.12)	*	0.15 (0.02, 1.12)	21	0.66 (0.40, 1.08)
	0 day	0	n/a	0	n/a			0	n/a	0	n/a	0	n/a
	1-7 days	9	0.99 (0.49, 2.00)	9	1.01 (0.50, 2.05)			9	1.00 (0.49, 2.02)	*	1.50 (0.43, 5.19)	9	0.97 (0.48, 1.96)
	8-14 days	7	0.71 (0.32, 1.56)	7	0.73 (0.33, 1.59)			7	0.72 (0.33, 1.57)	*	1.43 (0.41, 4.93)	7	0.70 (0.32, 1.53)
	15-21 days	9	0.91 (0.45, 1.84)	9	0.94 (0.47, 1.89)			9	0.92 (0.46, 1.85)	*	0.94 (0.22, 4.08)	9	0.90 (0.45, 1.80)
	22-28 days	9	0.90 (0.45, 1.82)	8	0.83 (0.40, 1.73)			9	0.91 (0.45, 1.82)	*	1.72 (0.57, 5.14)	9	0.89 (0.44, 1.78)
**Myasthenic disorder**													
**ChAdOx1nCoV-19 vaccine**	Baseline	311	1.00	302	1.00	133	1.00	309	1.00	69	1.00	309	1.00
	-28 to -1 days	70	0.72 (0.54, 0.96)	69	0.74 (0.55, 0.99)			70	0.72 (0.54, 0.96)	12	0.71 (0.37, 1.35)	70	0.73 (0.54, 0.97)
	0 day	0	n/a	0	n/a	0	n/a	0	n/a	0	n/a	0	n/a
	1-7 days	17	0.70 (0.42, 1.16)	15	0.65 (0.38, 1.12)	17	0.90 (0.47, 1.72)	17	0.70 (0.42, 1.16)	*	0.44 (0.10, 1.89)	17	0.70 (0.42, 1.17)
	8-14 days	26	1.16 (0.76, 1.77)	23	1.10 (0.70, 1.71)	26	1.37 (0.80, 2.35)	26	1.17 (0.76, 1.78)	8	1.88 (0.86, 4.14)	26	1.17 (0.77, 1.79)
	15-21 days	33	1.57 (1.07, 2.30)	33	1.66 (1.13, 2.43)	33	1.77 (1.11, 2.83)	33	1.58 (1.08, 2.31)	6	1.71 (0.72, 4.10)	33	1.58 (1.08, 2.32)
	22-28 days	23	1.01 (0.65, 1.57)	23	1.06 (0.68, 1.64)	23	1.12 (0.69, 1.83)	23	1.01 (0.65, 1.57)	5	1.40 (0.54, 3.61)	23	1.01 (0.65, 1.58)
	Baseline	213	1.00	205	1.00	132	1.00	213	1.00	38	1.00	211	1.00

**BNT162b2 mRNA vaccine**	-28 to -1 days	44	0.58 (0.41, 0.83)	44	0.61 (0.43, 0.86)			44	0.58 (0.41, 0.82)	8	0.92 (0.41, 2.06)	44	0.57 (0.41, 0.81)
	0 day	*	0.96 (0.30, 3.04)	*	1.01 (0.32, 3.20)	*	1.33 (0.37, 4.85)	*	0.96 (0.30, 3.03)	0	n/a	*	0.94 (0.30, 2.98)
	1-7 days	18	0.83 (0.50, 1.37)	16	0.79 (0.46, 1.34)	18	1.04 (0.50, 2.13)	18	0.83 (0.50, 1.37)	*	0.98 (0.28, 3.43)	18	0.81 (0.49, 1.34)
	8-14 days	24	1.09 (0.70, 1.71)	23	1.13 (0.71, 1.78)	24	1.21 (0.65, 2.24)	24	1.09 (0.70, 1.71)	*	1.09 (0.31, 3.76)	24	1.07 (0.68, 1.68)
	15-21 days	22	1.02 (0.64, 1.63)	20	1.01 (0.62, 1.63)	22	1.14 (0.64, 2.01)	22	1.02 (0.64, 1.62)	6	2.21 (0.84, 5.81)	22	1.01 (0.63, 1.60)
	22-28 days	27	1.46 (0.96, 2.22)	25	1.46 (0.95, 2.25)	27	1.61 (0.99, 2.64)	27	1.46 (0.96, 2.21)	5	2.35 (0.85, 6.46)	27	1.45 (0.96, 2.20)
**Haemorrhagic stroke**													
**ChAdOx1nCoV-19 vaccine**	Baseline	1456	1.00	1195	1.00	668	1.00	1424	1.00	750	1.00	1436	1.00
	-28 to -1 days	232	0.47 (0.41, 0.55)	224	0.57 (0.49, 0.67)			232	0.48 (0.41, 0.55)	61	0.26 (0.20, 0.33)	232	0.49 (0.42, 0.56)
	0 day	7	0.37 (0.18, 0.78)	6	0.41 (0.19, 0.93)	7	0.22 (0.10, 0.47)	7	0.37 (0.18, 0.78)	*	0.42 (0.16, 1.13)	7	0.38 (0.18, 0.80)
	1-7 days	131	0.98 (0.82, 1.18)	94	0.93 (0.75, 1.15)	131	0.61 (0.48, 0.78)	130	0.98 (0.81, 1.18)	91	1.35 (1.07, 1.69)	131	1.01 (0.84, 1.21)
	8-14 days	141	1.06 (0.89, 1.27)	93	0.93 (0.75, 1.16)	141	0.72 (0.57, 0.90)	139	1.06 (0.88, 1.27)	104	1.53 (1.24, 1.90)	141	1.08 (0.91, 1.30)
	15-21 days	128	0.98 (0.81, 1.18)	92	0.95 (0.77, 1.19)	128	0.72 (0.58, 0.89)	127	0.98 (0.81, 1.18)	85	1.25 (0.99, 1.58)	128	0.99 (0.82, 1.19)
	22-28 days	126	1.00 (0.83, 1.21)	86	0.95 (0.76, 1.19)	126	0.79 (0.64, 0.97)	125	1.00 (0.83, 1.21)	62	0.94 (0.72, 1.23)	126	1.01 (0.83, 1.21)
**BNT162b2 mRNA vaccine**	Baseline	811	1.00	574	1.00	622	1.00	806	1.00	416	1.00	800	1.00
	-28 to -1 days	86	0.36 (0.29, 0.46)	83	0.43 (0.34, 0.55)			84	0.35 (0.28, 0.44)	13	0.18 (0.10, 0.32)	86	0.34 (0.27, 0.43)
	0 day	6	0.59 (0.27, 1.33)	*	0.39 (0.12, 1.21)	6	0.26 (0.11, 0.60)	6	0.59 (0.26, 1.31)	*	0.87 (0.28, 2.73)	6	0.56 (0.25, 1.26)
	1-7 days	93	1.27 (1.02, 1.59)	63	1.14 (0.87, 1.50)	93	0.60 (0.44, 0.82)	93	1.25 (1.00, 1.57)	51	1.94 (1.42, 2.66)	93	1.21 (0.96, 1.51)
	8-14 days	89	1.18 (0.94, 1.48)	59	1.06 (0.80, 1.40)	89	0.64 (0.48, 0.86)	89	1.16 (0.92, 1.46)	52	1.85 (1.36, 2.51)	89	1.12 (0.89, 1.40)

	15-21 days	106	1.38 (1.12, 1.71)	69	1.24 (0.96, 1.61)	106	0.86 (0.67, 1.11)	106	1.37 (1.11, 1.69)	64	2.13 (1.61, 2.81)	106	1.31 (1.06, 1.62)
	22-28 days	91	1.15 (0.92, 1.44)	59	1.06 (0.81, 1.40)	91	0.80 (0.62, 1.02)	91	1.14 (0.91, 1.42)	60	1.79 (1.35, 2.38)	91	1.09 (0.87, 1.36)
**Subarachnoid haemorrhage**													
**ChAdOx1nCoV-19 vaccine**	Baseline	971	1.00	866	1.00	343	1.00	954	1.00	460	1.00	966	1.00
	-28 to -1 days	157	0.49 (0.41, 0.58)	155	0.55 (0.46, 0.65)			157	0.49 (0.41, 0.58)	37	0.22 (0.16, 0.31)	157	0.49 (0.41, 0.58)
	0 day	*	0.34 (0.13, 0.91)	*	0.20 (0.05, 0.80)	*	0.26 (0.09, 0.71)	*	0.34 (0.13, 0.91)	*	0.66 (0.24, 1.76)	*	0.34 (0.13, 0.92)
	1-7 days	70	0.85 (0.66, 1.10)	56	0.81 (0.61, 1.07)	70	0.65 (0.46, 0.92)	70	0.86 (0.67, 1.10)	44	1.06 (0.77, 1.47)	70	0.86 (0.67, 1.10)
	8-14 days	93	1.16 (0.93, 1.44)	76	1.14 (0.89, 1.45)	93	0.93 (0.69, 1.26)	91	1.14 (0.91, 1.42)	63	1.61 (1.22, 2.13)	93	1.16 (0.93, 1.45)
	15-21 days	72	0.92 (0.72, 1.18)	52	0.82 (0.61, 1.09)	72	0.77 (0.57, 1.04)	72	0.92 (0.72, 1.19)	54	1.44 (1.07, 1.94)	72	0.92 (0.72, 1.18)
	22-28 days	69	0.94 (0.73, 1.20)	49	0.82 (0.61, 1.10)	69	0.82 (0.62, 1.09)	69	0.94 (0.73, 1.21)	44	1.26 (0.91, 1.74)	69	0.93 (0.72, 1.19)
**BNT162b2 mRNA vaccine**	Baseline	415	1.00	355	1.00	240	1.00	409	1.00	191	1.00	405	1.00
	-28 to -1 days	53	0.43 (0.32, 0.58)	53	0.47 (0.35, 0.64)			53	0.44 (0.32, 0.59)	*	0.10 (0.03, 0.30)	53	0.42 (0.31, 0.56)
	0 day	0	n/a	0	n/a	0	n/a	0	n/a	0	n/a	0	n/a
	1-7 days	38	1.08 (0.77, 1.52)	25	0.81 (0.53, 1.22)	38	0.85 (0.53, 1.37)	38	1.09 (0.77, 1.54)	21	2.17 (1.35, 3.50)	38	1.05 (0.75, 1.48)
	8-14 days	41	1.14 (0.82, 1.59)	37	1.20 (0.84, 1.70)	41	0.93 (0.61, 1.42)	41	1.15 (0.82, 1.60)	15	1.43 (0.83, 2.47)	41	1.11 (0.80, 1.55)
	15-21 days	31	0.85 (0.58, 1.23)	24	0.77 (0.50, 1.17)	31	0.74 (0.48, 1.14)	31	0.85 (0.59, 1.24)	14	1.28 (0.73, 2.25)	31	0.83 (0.57, 1.20)
	22-28 days	41	1.16 (0.84, 1.61)	32	1.08 (0.75, 1.56)	41	1.02 (0.71, 1.47)	41	1.17 (0.84, 1.62)	21	1.82 (1.14, 2.91)	41	1.14 (0.82, 1.58)
**Celiac**													
**ChAdOx1nCoV-19 vaccine**	Baseline	2403	1.00	2400	1.00	775	1.00	2368	1.00	594	1.00	2399	1.00
	-28 to -1 days	715	0.95 (0.87, 1.04)	715	0.95 (0.87, 1.04)			715	0.95 (0.87, 1.04)	174	0.79 (0.65, 0.96)	715	0.95 (0.86, 1.04)

	0 day	*	0.07 (0.02, 0.29)	*	0.07 (0.02, 0.29)	*	0.10 (0.02, 0.39)	*	0.07 (0.02, 0.29)	0	n/a	*	0.07 (0.02, 0.29)
	1-7 days	164	0.84 (0.72, 0.99)	164	0.84 (0.72, 0.99)	164	1.11 (0.88, 1.40)	164	0.85 (0.72, 1.00)	38	0.74 (0.52, 1.06)	164	0.84 (0.71, 0.99)
	8-14 days	196	1.01 (0.87, 1.17)	196	1.01 (0.87, 1.18)	196	1.28 (1.04, 1.58)	196	1.02 (0.87, 1.18)	38	0.79 (0.56, 1.12)	196	1.01 (0.87, 1.17)
	15-21 days	184	0.97 (0.83, 1.13)	184	0.97 (0.83, 1.13)	184	1.19 (0.98, 1.45)	184	0.97 (0.83, 1.14)	36	0.79 (0.55, 1.14)	184	0.97 (0.83, 1.13)
	22-28 days	183	0.99 (0.85, 1.16)	183	0.99 (0.85, 1.16)	183	1.19 (0.99, 1.43)	183	1.00 (0.85, 1.16)	38	0.94 (0.66, 1.33)	183	0.99 (0.85, 1.15)
**BNT162b2 mRNA vaccine**	Baseline	1610	1.00	1606	1.00	755	1.00	1597	1.00	279	1.00	1595	1.00
	-28 to -1 days	437	0.88 (0.79, 0.98)	437	0.88 (0.79, 0.99)			437	0.88 (0.79, 0.99)	29	0.82 (0.55, 1.22)	437	0.88 (0.79, 0.98)
	0 day	5	0.27 (0.11, 0.65)	5	0.27 (0.11, 0.65)	5	0.38 (0.15, 0.93)	5	0.27 (0.11, 0.65)	*	1.00 (0.14, 7.19)	5	0.27 (0.11, 0.65)
	1-7 days	98	0.75 (0.61, 0.92)	98	0.75 (0.61, 0.93)	98	1.01 (0.76, 1.34)	97	0.75 (0.60, 0.92)	12	1.77 (0.97, 3.24)	98	0.75 (0.61, 0.92)
	8-14 days	121	0.90 (0.75, 1.09)	121	0.91 (0.75, 1.10)	121	1.14 (0.89, 1.46)	121	0.91 (0.75, 1.10)	15	2.17 (1.25, 3.76)	121	0.90 (0.75, 1.09)
	15-21 days	122	0.89 (0.73, 1.07)	119	0.87 (0.72, 1.05)	122	1.11 (0.88, 1.40)	122	0.89 (0.74, 1.08)	14	2.04 (1.15, 3.61)	122	0.89 (0.73, 1.07)
	22-28 days	116	0.86 (0.71, 1.05)	116	0.87 (0.71, 1.05)	116	1.02 (0.82, 1.27)	116	0.86 (0.71, 1.05)	7	1.05 (0.48, 2.28)	116	0.86 (0.71, 1.04)
**Anaphylaxis**													
**ChAdOx1nCoV-19 vaccine**	Baseline	438	1.00	438	1.00	160	1.00	422	1.00	164	1.00	433	1.00
	-28 to -1 days	111	0.82 (0.66, 1.02)	111	0.82 (0.66, 1.02)			111	0.83 (0.66, 1.03)	39	0.81 (0.55, 1.18)	111	0.82 (0.66, 1.02)
	0 day	47	9.68 (7.08, 13.24)	47	9.72 (7.10, 13.29)	47	8.33 (4.93, 14.08)	46	9.64 (7.02, 13.24)	41	29.79 (20.33, 43.63)	47	9.71 (7.10, 13.28)
	1-7 days	52	1.55 (1.15, 2.09)	52	1.55 (1.15, 2.10)	52	1.36 (0.83, 2.22)	52	1.57 (1.16, 2.12)	27	2.97 (1.91, 4.63)	52	1.55 (1.15, 2.10)
	8-14 days	25	0.74 (0.49, 1.11)	25	0.74 (0.49, 1.11)	25	0.64 (0.37, 1.08)	25	0.75 (0.50, 1.13)	8	0.88 (0.42, 1.84)	25	0.74 (0.49, 1.11)
	15-21 days	36	1.05	36	1.05	36	0.94	36	1.07	12	1.34	36	1.05

			(0.74, 1.49)		(0.74, 1.49)		(0.60, 1.46)		(0.75, 1.52)		(0.72, 2.49)		(0.74, 1.49)
	22-28 days	26	0.78 (0.52, 1.18)	26	0.79 (0.52, 1.18)	26	0.73 (0.46, 1.16)	26	0.80 (0.53, 1.20)	*	0.50 (0.18, 1.37)	26	0.78 (0.52, 1.17)
**BNT162b2 mRNA vaccine**	Baseline	167	1.00	165	1.00	76	1.00	162	1.00	64	1.00	165	1.00
	-28 to -1 days	31	0.73 (0.49, 1.08)	31	0.74 (0.50, 1.10)			31	0.77 (0.51, 1.14)	8	0.79 (0.37, 1.69)	31	0.71 (0.48, 1.06)
	0 day	29	19.31 (12.77, 29.19)	29	19.65 (12.99, 29.72)	29	14.81 (7.51, 29.21)	29	19.92 (13.13, 30.22)	23	83.74 (47.35, 148.08)	29	18.78 (12.42, 28.39)
	1-7 days	16	1.50 (0.89, 2.55)	16	1.53 (0.90, 2.60)	16	1.22 (0.59, 2.50)	16	1.54 (0.91, 2.62)	9	4.54 (2.11, 9.77)	16	1.46 (0.86, 2.48)
	8-14 days	22	1.99 (1.25, 3.17)	22	2.03 (1.28, 3.23)	22	1.63 (0.87, 3.04)	21	1.94 (1.21, 3.12)	5	2.38 (0.90, 6.31)	22	1.94 (1.22, 3.08)
	15-21 days	12	1.10 (0.60, 2.00)	12	1.12 (0.61, 2.04)	12	1.01 (0.51, 2.01)	12	1.12 (0.61, 2.04)	*	2.12 (0.73, 6.13)	12	1.07 (0.59, 1.95)
	22-28 days	11	0.99 (0.53, 1.84)	11	1.00 (0.54, 1.87)	11	0.94 (0.48, 1.87)	10	0.92 (0.48, 1.76)	*	1.02 (0.24, 4.35)	11	0.96 (0.51, 1.79)

*****Sensitivity 1: excluding those who died from the outcome; Sensitivity 2: restricting analysis to the period post-vaccination, without censoring at death, Sensitivity 3: restricting the study period until the 1 May 2021, Sensitivity 4: restricting analysis to patients who received only one vaccine dose and Sensitivity 5: censoring at 12 weeks post vaccination.

° Sensitivity model 2 does not converge.

Supplementary table 7b: Incidence rate ratios (IRR 95% CI) for single outcomes in pre-defined risk periods 28 days immediately before and 35 days after exposure to vaccination and to a positive SARS-CoV-2 test, adjusted for calendar time from 1 December 2020 to 31 May 2021.

		ChAdOx1nCoV-19 vaccine		BNT162b2 mRNA vaccine		Positive SARS-CoV-2 test	
	**Time period**	**events**	**IRR (95% CI)**	**events**	**IRR (95% CI)**	**events**	**IRR (95% CI)**
**CNS Demyelinating disorder**	Baseline	440	1.00	230	1.00	52	1.00
	-28 to -1 days	117	0.82 (0.66, 1.02)	58	0.90 (0.66, 1.22)	27	3.04 (1.83, 5.04)
	0 day	0	n/a	*	0.42 (0.06, 2.99)	7	19.97 (8.86, 45.01)
	1-7 days	38	1.02 (0.72, 1.44)	15	0.89 (0.52, 1.52)	9	3.56 (1.71, 7.40)

	8-14 days	35	0.95 (0.66, 1.36)	16	0.92 (0.55, 1.55)	7	2.70 (1.20, 6.06)
	15-21 days	38	1.03 (0.73, 1.46)	16	0.90 (0.54, 1.52)	6	2.11 (0.90, 4.99)
	22-28 days	33	0.93 (0.64, 1.34)	21	1.16 (0.73, 1.84)	*	0.62 (0.15, 2.55)
	29-35 days	33	1.00 (0.69, 1.45)	14	0.78 (0.45, 1.35)	5	1.46 (0.58, 3.67)
**Encephalitis/meningitis/myelitis**	Baseline	569	1.00	247	1.00	65	1.00
	-28 to -1 days	78	0.42 (0.33, 0.54)	45	0.62 (0.44, 0.86)	78	5.78 (3.97, 8.41)
	0 day	*	0.46 (0.15, 1.43)	0	n/a	23	39.70 (23.93, 65.86)
	1-7 days	52	1.15 (0.85, 1.55)	22	1.08 (0.68, 1.70)	24	5.87 (3.57, 9.65)
	8-14 days	58	1.33 (0.99, 1.77)	27	1.24 (0.81, 1.88)	24	5.80 (3.54, 9.51)
	15-21 days	37	0.89 (0.63, 1.26)	21	1.01 (0.63, 1.60)	6	1.41 (0.60, 3.30)
	22-28 days	38	0.96 (0.68, 1.36)	27	1.35 (0.89, 2.05)	6	1.37 (0.59, 3.19)
	29-35 days	33	0.92 (0.64, 1.33)	28	1.45 (0.97, 2.18)	6	1.37 (0.59, 3.19)
**Guillain-Barre**	Baseline	235	1.00	97	1.00	25	1.00
	-28 to -1 days	41	0.47 (0.33, 0.67)	21	0.68 (0.41, 1.13)	23	5.85 (3.12, 10.96)
	0 day	0	n/a	0	n/a	7	40.39 (16.84, 96.84)
	1-7 days	17	0.80 (0.48, 1.34)	9	1.03 (0.51, 2.10)	11	8.97 (4.24, 18.94)
	8-14 days	23	1.12 (0.71, 1.77)	7	0.75 (0.34, 1.65)	8	6.33 (2.76, 14.52)
	15-21 days	65	3.20 (2.34, 4.37)	9	0.97 (0.48, 1.97)	11	8.44 (4.03, 17.68)
	22-28 days	48	2.44 (1.73, 3.44)	9	0.97 (0.48, 1.96)	6	4.29 (1.72, 10.68)
	29-35 days	29	1.55 (1.03, 2.34)	12	1.32 (0.71, 2.46)	6	4.03 (1.63, 9.98)
**Bell's palsy**	Baseline	1248	1.00	655	1.00	138	1.00
	-28 to -1 days	328	0.81 (0.71, 0.92)	168	0.77 (0.64, 0.92)	86	3.34 (2.48, 4.49)
	0 day	5	0.34 (0.14, 0.81)	*	0.36 (0.12, 1.12)	35	33.23 (22.50, 49.09)
	1-7 days	95	0.90 (0.73, 1.12)	54	0.90 (0.68, 1.20)	44	5.84 (4.08, 8.36)
	8-14 days	84	0.80 (0.64, 1.00)	57	0.94 (0.71, 1.24)	17	2.17 (1.30, 3.64)

	15-21 days	136	1.31 (1.09, 1.58)	65	1.04 (0.80, 1.36)	9	1.09 (0.55, 2.15)
	22-28 days	115	1.14 (0.94, 1.39)	71	1.16 (0.90, 1.49)	7	0.80 (0.37, 1.72)
	29-35 days	108	1.13 (0.92, 1.38)	57	0.92 (0.70, 1.22)	9	0.96 (0.49, 1.89)
**Myasthenic disorder**	Baseline	292	1.00	199	1.00	30	1.00
	-28 to -1 days	70	0.70 (0.52, 0.93)	44	0.57 (0.40, 0.81)	26	4.13 (2.34, 7.27)
	0 day	0	n/a	*	0.94 (0.30, 2.96)	18	62.69 (33.79, 116.30)
	1-7 days	17	0.67 (0.40, 1.12)	18	0.80 (0.48, 1.34)	29	14.05 (8.14, 24.25)
	8-14 days	26	1.12 (0.73, 1.72)	24	1.06 (0.67, 1.66)	9	4.09 (1.90, 8.82)
	15-21 days	33	1.51 (1.03, 2.23)	22	0.99 (0.62, 1.57)	5	2.23 (0.85, 5.83)
	22-28 days	23	0.97 (0.62, 1.52)	27	1.40 (0.92, 2.14)	6	2.89 (1.19, 7.05)
	29-35 days	19	0.81 (0.50, 1.30)	14	0.73 (0.42, 1.27)	*	1.47 (0.45, 4.86)
**Haemorrhagic stroke**	Baseline	1325	1.00	724	1.00	165	1.00
	-28 to -1 days	232	0.48 (0.42, 0.56)	86	0.36 (0.29, 0.46)	163	4.79 (3.74, 6.12)
	0 day	7	0.38 (0.18, 0.80)	6	0.60 (0.27, 1.35)	20	12.58 (7.81, 20.26)
	1-7 days	131	1.00 (0.83, 1.21)	93	1.29 (1.03, 1.62)	23	2.04 (1.30, 3.20)
	8-14 days	141	1.09 (0.91, 1.31)	89	1.19 (0.95, 1.50)	14	1.21 (0.69, 2.10)
	15-21 days	128	1.00 (0.83, 1.21)	106	1.41 (1.14, 1.74)	10	0.86 (0.45, 1.63)
	22-28 days	126	1.03 (0.85, 1.24)	91	1.17 (0.94, 1.47)	6	0.52 (0.23, 1.17)
	29-35 days	131	1.14 (0.95, 1.38)	87	1.11 (0.88, 1.39)	13	1.10 (0.62, 1.95)
**Subarachnoid haemorrhage**	Baseline	910	1.00	385	1.00	93	1.00
	-28 to -1 days	157	0.48 (0.40, 0.57)	53	0.43 (0.32, 0.57)	60	3.22 (2.24, 4.61)
	0 day	*	0.33 (0.13, 0.90)	0	n/a	19	22.90 (13.69, 38.31)
	1-7 days	70	0.84 (0.65, 1.08)	38	1.06 (0.75, 1.49)	23	3.93 (2.44, 6.35)
	8-14 days	93	1.13 (0.90, 1.41)	41	1.11 (0.80, 1.55)	12	2.03 (1.10, 3.77)
	15-21 days	72	0.90 (0.70, 1.16)	31	0.83 (0.57, 1.20)	7	1.16 (0.53, 2.53)
	22-28 days	69	0.91 (0.71, 1.18)	41	1.13 (0.81, 1.57)	7	1.13 (0.52, 2.45)

	29-35 days	61	0.87 (0.67, 1.14)	30	0.83 (0.57, 1.21)	*	0.31 (0.08, 1.25)

Supplementary table 7c: Incidence rate ratios (IRR 95% CI) for individual outcomes in pre-defined risk periods immediately before and after exposure to a positive SARS-CoV-2 test result, adjusted for calendar time from 1 December 2020 until 31 May 2021, 2021 (cells with < 5 are suppressed): comparisons between patients infected before vaccination and all patients (infected before and after vaccination)

		Positive SARS-CoV-2 test (before vaccination)		Positive SARS-CoV-2 test before or after vaccination)	
	Time period	events	IRR (95% CI)	events	IRR (95% CI)
**Acute CNS Demyelinating disorder**	Baseline	52	1.00	53	1.00
	-28 to -1 days	21	2.35 (1.37, 4.03)	27	2.94 (1.79, 4.83)
	0 day	6	17.12 (7.22, 40.62)	7	19.34 (8.63, 43.38)
	1-7 days	7	2.78 (1.24, 6.22)	9	3.45 (1.67, 7.14)
	8-14 days	7	2.70 (1.21, 6.04)	7	2.61 (1.17, 5.84)
	15-21 days	6	2.12 (0.90, 4.97)	6	2.05 (0.87, 4.82)
	22-28 days	*	0.62 (0.15, 2.54)	*	0.60 (0.15, 2.47)
**Encephalitis meningitis and myelitis**	Baseline	55	1.00	66	1.00
	-28 to -1 days	63	6.14 (4.07, 9.26)	78	5.64 (3.91, 8.13)
	0 day	16	36.22 (20.15, 65.09)	23	38.57 (23.41, 63.56)
	1-7 days	18	5.79 (3.31, 10.14)	24	5.71 (3.49, 9.32)
	8-14 days	22	6.81 (4.05, 11.44)	24	5.63 (3.46, 9.15)

	15-21 days	6	1.81 (0.77, 4.26)	6	1.38 (0.59, 3.21)
	22-28 days	5	1.43 (0.57, 3.58)	6	1.33 (0.57, 3.08)
**Guillain-Barre syndrome**	Baseline	28	1.00	30	1.00
	-28 to -1 days	20	5.14 (2.74, 9.67)	23	4.85 (2.67, 8.82)
	0 day	5	28.47 (10.67, 75.96)	7	33.37 (14.21, 78.36)
	1-7 days	8	6.39 (2.82, 14.47)	11	7.36 (3.57, 15.18)
	8-14 days	8	6.25 (2.76, 14.13)	8	5.19 (2.31, 11.65)
	15-21 days	10	7.30 (3.45, 15.42)	11	6.89 (3.37, 14.09)
	22-28 days	6	4.02 (1.64, 9.86)	6	3.51 (1.44, 8.57)
**Bell's palsy**	Baseline	120	1.00	134	1.00
	-28 to -1 days	65	3.09 (2.22, 4.29)	86	3.34 (2.49, 4.48)
	0 day	27	31.04 (20.09, 47.94)	35	33.23 (22.57, 48.94)
	1-7 days	35	5.66 (3.82, 8.40)	44	5.84 (4.09, 8.33)
	8-14 days	15	2.36 (1.37, 4.08)	17	2.17 (1.30, 3.63)
	15-21 days	8	1.18 (0.57, 2.42)	9	1.09 (0.55, 2.15)
	22-28 days	5	0.68 (0.28, 1.67)	7	0.80 (0.37, 1.72)
	Baseline	28	1.00	32	1.00

**Myasthenic disorder**	-28 to -1 days	19	3.59 (1.92, 6.73)	26	4.00 (2.30, 6.96)
	0 day	13	54.96 (27.50, 109.82)	18	61.32 (33.43, 112.50)
	1-7 days	22	13.22 (7.30, 23.97)	29	13.74 (8.07, 23.41)
	8-14 days	8	4.51 (2.01, 10.14)	9	3.98 (1.86, 8.52)
	15-21 days	*	1.65 (0.49, 5.48)	5	2.17 (0.84, 5.63)
	22-28 days	5	2.92 (1.11, 7.64)	6	2.81 (1.16, 6.79)
**Haemorrhagic stroke**	Baseline	149	1.00	173	1.00
	-28 to -1 days	125	4.75 (3.62, 6.24)	163	4.73 (3.72, 6.02)
	0 day	13	10.90 (6.11, 19.46)	20	12.42 (7.73, 19.95)
	1-7 days	14	1.64 (0.94, 2.87)	23	2.01 (1.29, 3.15)
	8-14 days	6	0.67 (0.30, 1.54)	14	1.19 (0.69, 2.07)
	15-21 days	6	0.66 (0.29, 1.51)	10	0.84 (0.44, 1.61)
	22-28 days	5	0.55 (0.23, 1.36)	6	0.51 (0.23, 1.16)
**Subarachnoid haemorrhage**	Baseline	75	1.00	88	1.00
	-28 to -1 days	51	3.64 (2.46, 5.39)	60	3.40 (2.38, 4.86)
	0 day	12	19.89 (10.61, 37.28)	19	24.22 (14.50, 40.45)

	1-7 days	16	3.81 (2.18, 6.67)	23	4.17 (2.59, 6.71)
	8-14 days	8	1.88 (0.90, 3.95)	12	2.15 (1.16, 3.99)
	15-21 days	6	1.38 (0.60, 3.20)	7	1.23 (0.57, 2.67)
	22-28 days	6	1.32 (0.57, 3.05)	7	1.19 (0.55, 2.58)

Supplementary table 7d: Incidence rate ratios (IRR 95% CI) for single outcomes in pre-defined risk periods 28 days immediately before and 28 days after exposure to vaccination and to a positive SARS-CoV-2 test, adjusted for calendar time from 1 December 2020 to 31 May 2021 restricted to patients who did not have a SARS-CoV-2 positive test.

		ChAdOx1nCoV-19 vaccine		BNT162b2 mRNA vaccine	
	**Time period**	**events**	**IRR (95% CI)**	**events**	**IRR (95% CI)**
**CNS Demyelinating disorder**	Baseline	406	1.00	221	1.00
	-28 to -1 days	108	0.88 (0.70, 1.11)	53	0.92 (0.67, 1.25)
	0 day	0	n/a	*	0.48 (0.07, 3.44)
	1-7 days	36	1.10 (0.77, 1.56)	12	0.83 (0.46, 1.49)
	8-14 days	31	0.96 (0.66, 1.40)	12	0.80 (0.44, 1.45)
	15-21 days	31	0.96 (0.66, 1.41)	15	0.97 (0.57, 1.65)
	22-28 days	30	0.96 (0.65, 1.40)	18	1.14 (0.69, 1.86)
**Encephalitis/meningitis/myelitis**	Baseline	455	1.00	232	1.00
	-28 to -1 days	62	0.39 (0.30, 0.52)	37	0.58 (0.40, 0.83)
	0 day	*	0.52 (0.17, 1.62)	0	n/a
	1-7 days	43	1.08 (0.78, 1.50)	19	1.05 (0.65, 1.71)
	8-14 days	52	1.33 (0.98, 1.79)	21	1.11 (0.70, 1.77)
	15-21 days	30	0.79 (0.54, 1.16)	20	1.07 (0.67, 1.71)
	22-28 days	34	0.95 (0.66, 1.36)	22	1.20 (0.76, 1.88)
**Guillain-Barre**	Baseline	201	1.00	88	1.00

	-28 to -1 days	33	0.46 (0.31, 0.68)	20	0.86 (0.51, 1.43)
	0 day	0	n/a	0	n/a
	1-7 days	12	0.59 (0.32, 1.07)	8	1.15 (0.55, 2.43)
	8-14 days	21	1.03 (0.64, 1.64)	5	0.66 (0.26, 1.65)
	15-21 days	63	3.06 (2.24, 4.18)	8	1.00 (0.48, 2.11)
	22-28 days	46	2.32 (1.64, 3.27)	7	0.85 (0.39, 1.87)
**Bell's palsy**	Baseline	1156	1.00	646	1.00
	-28 to -1 days	295	0.81 (0.71, 0.93)	157	0.81 (0.67, 0.97)
	0 day	5	0.37 (0.15, 0.89)	*	0.40 (0.13, 1.26)
	1-7 days	80	0.84 (0.67, 1.06)	47	0.88 (0.65, 1.20)
	8-14 days	73	0.77 (0.60, 0.98)	51	0.94 (0.70, 1.27)
	15-21 days	126	1.33 (1.10, 1.61)	59	1.07 (0.81, 1.40)
	22-28 days	99	1.06 (0.86, 1.31)	61	1.11 (0.85, 1.45)
**Myasthenic disorder**	Baseline	232	1.00	191	1.00
	-28 to -1 days	62	0.83 (0.61, 1.14)	40	0.60 (0.42, 0.86)
	0 day	0	n/a	*	1.08 (0.34, 3.41)
	1-7 days	15	0.78 (0.45, 1.34)	15	0.82 (0.48, 1.42)
	8-14 days	24	1.32 (0.85, 2.06)	20	1.09 (0.67, 1.77)
	15-21 days	29	1.64 (1.09, 2.47)	18	0.99 (0.60, 1.64)
	22-28 days	20	1.04 (0.65, 1.67)	25	1.54 (1.00, 2.38)
**Haemorrhagic stroke**	Baseline	1169	1.00	746	1.00
	-28 to -1 days	214	0.51 (0.44, 0.59)	81	0.38 (0.30, 0.49)
	0 day	6	0.37 (0.16, 0.82)	5	0.56 (0.23, 1.35)
	1-7 days	112	0.97 (0.80, 1.19)	82	1.27 (1.00, 1.61)
	8-14 days	123	1.06 (0.88, 1.29)	74	1.08 (0.84, 1.38)

	15-21 days	114	0.98 (0.81, 1.20)	96	1.36 (1.09, 1.70)
	22-28 days	116	1.03 (0.85, 1.26)	82	1.12 (0.88, 1.41)
**Subarachnoid haemorrhage**	Baseline	833	1.00	374	1.00
	-28 to -1 days	136	0.47 (0.39, 0.57)	49	0.44 (0.32, 0.59)
	0 day	*	0.19 (0.05, 0.76)	0	n/a
	1-7 days	66	0.91 (0.70, 1.17)	31	0.98 (0.68, 1.43)
	8-14 days	76	1.06 (0.83, 1.35)	37	1.17 (0.82, 1.65)
	15-21 days	62	0.88 (0.67, 1.15)	26	0.80 (0.53, 1.20)
	22-28 days	63	0.94 (0.72, 1.22)	38	1.18 (0.84, 1.66)

Supplementary table 8: ICD-10 codes used to identify cases with each outcome.

**Composite outcomes**	**ICD-10 code and description**
**Demyelinating disorder**	G361 Acute and subacute haemorrhagic leukoencephalitis [Hurst]<br>G368 Other specified acute disseminated demyelination<br>G369 Acute disseminated demyelination, unspecified<br>G372 Central pontine myelinolysis<br>G373 Acute transverse myelitis in demyelinating disease of central nervous system<br>G378 Other specified demyelinating diseases of central nervous system<br>G379 Demyelinating disease of central nervous system, unspecified<br>H46X Optic neuritis<br>H481 Retrobulbar neuritis in diseases classified elsewhere
	A87 - Viral meningitis

**Encephalitis meningitis and myelitis**	A870 - Enteroviral meningitis
	A871 - Adenoviral meningitis
	A878 - Other viral meningitis
	A879 - Viral meningitis, unspecified
	G030 - Nonpyogenic meningitis
	G039 - Meningitis, unspecified
	G04 - Encephalitis, myelitis and encephalomyelitis
	G048 - Other encephalitis, myelitis and encephalomyelitis
	G049 - Encephalitis, myelitis and encephalomyelitis, unspecified
	G05 – Encephalitis, myelitis, encephalomyelitis in diseases classified elsewhere
	G051 - Encephalitis, myelitis and encephalomyelitis in viral diseases classified elsewher
	G058 - Encephalitis, myelitis and encephalomyelitis in other diseases classified elsewhere
**Guillain-Barre**	G610 - Guillain-Barré syndrome
**Bell's palsy**	G510 - Bell palsy
**Myasthenic disorder**	G70 - Myasthenia gravis and other myoneural disorders
	G700 - Myasthenia gravis
	G709 - Myoneural disorder, unspecified
	G733 - Myasthenic syndromes in other diseases classified elsewhere
**Haemorrhagic stroke**	I61 - Intracerebral haemorrhage

	I610 - Intracerebral haemorrhage in hemisphere, subcortical
	I611, I611 - Intracerebral haemorrhage in hemisphere, cortical
	I612, I612 - Intracerebral haemorrhage in hemisphere, unspecified
	I613, I613 - Intracerebral haemorrhage in brain stem
	I614, I614 - Intracerebral haemorrhage in cerebellum
	I615, I615 - Intracerebral haemorrhage, intraventricular
	I616, I616 - Intracerebral haemorrhage, multiple localized
	I618, I618 - Other intracerebral haemorrhage
	I619, I619 - Intracerebral haemorrhage, unspecified
**Subarachnoid haemorrhage**	I60 - Subarachnoid haemorrhage
	I600 - Subarachnoid haemorrhage from carotid siphon and bifurcation
	I601 - Subarachnoid haemorrhage from middle cerebral artery
	I602 - Subarachnoid haemorrhage from anterior communicating artery
	I603 - Subarachnoid haemorrhage from posterior communicating artery
	I604 - Subarachnoid haemorrhage from basilar artery
	I605 - Subarachnoid haemorrhage from vertebral artery
	I606 - Subarachnoid haemorrhage from other intracranial arteries
	I607 - Subarachnoid haemorrhage from intracranial artery, unspecified
	I608 - Other subarachnoid haemorrhage
	I609 - Subarachnoid haemorrhage, unspecified

	I690 - Sequelae of subarachnoid haemorrhage
