# Supplementary material for: Neurological complications after first dose of COVID-19 vaccines and SARS-CoV-2 infection
Source: Nat Med. 2021 Oct 25;27(12):2144–53. doi: 10.1038/s41591-021-01556-7 (PMC8629105; doi:10.1038/s41591-021-01556-7)
Supplement: Supplementary file 2 — Reporting Summary [file 41591_2021_1556_MOESM2_ESM.pdf]

nature portfolio

Corresponding author(s): Julia Hippisley-Cox

Last updated by author(s): Sep 22, 2021

Reporting Summary

Nature Portfolio wishes to improve the reproducibility of the work that we publish. This form provides structure for consistency and transparency in reporting. For further information on Nature Portfolio policies, see our Editorial Policies and the Editorial Policy Checklist.

Statistics

For all statistical analyses, confirm that the following items are present in the figure legend, table legend, main text, or Methods section.

n/a	Confirmed	
☐	☒	The exact sample size (n) for each experimental group/condition, given as a discrete number and unit of measurement
☐	☒	A statement on whether measurements were taken from distinct samples or whether the same sample was measured repeatedly
☐	☒	The statistical test(s) used AND whether they are one- or two-sided<br>*Only common tests should be described solely by name; describe more complex techniques in the Methods section.*
☐	☒	A description of all covariates tested
☐	☒	A description of any assumptions or corrections, such as tests of normality and adjustment for multiple comparisons
☐	☒	A full description of the statistical parameters including central tendency (e.g. means) or other basic estimates (e.g. regression coefficient) AND variation (e.g. standard deviation) or associated estimates of uncertainty (e.g. confidence intervals)
☐	☒	For null hypothesis testing, the test statistic (e.g. F, t, r) with confidence intervals, effect sizes, degrees of freedom and P value noted<br>*Give P values as exact values whenever suitable.*
☒	☐	For Bayesian analysis, information on the choice of priors and Markov chain Monte Carlo settings
☒	☐	For hierarchical and complex designs, identification of the appropriate level for tests and full reporting of outcomes
☒	☐	Estimates of effect sizes (e.g. Cohen's d, Pearson's r), indicating how they were calculated

Our web collection on statistics for biologists contains articles on many of the points above.

Software and code

Policy information about availability of computer code

Data collection	No software was used
Data analysis	Stata version 17 was used for data analysis

For manuscripts utilizing custom algorithms or software that are central to the research but not yet described in published literature, software must be made available to editors and reviewers. We strongly encourage code deposition in a community repository (e.g. GitHub). See the Nature Portfolio guidelines for submitting code & software for further information.

Data

Policy information about availability of data

All manuscripts must include a data availability statement. This statement should provide the following information, where applicable:

- Accession codes, unique identifiers, or web links for publicly available datasets
- A description of any restrictions on data availability
- For clinical datasets or third party data, please ensure that the statement adheres to our policy

The data that support the findings of this study [National Immunisation (NIMS) Database of COVID-19, mortality (ONS), hospital admissions (HES) and SARS-CoV-2 infection data (PHE)] are not publicly available because they are based on de-identified national clinical records. To guarantee the confidentiality of personal and health information, only the authors had access to the data in accordance with relevant ethical and license agreements.

March 2021

Field-specific reporting

Please select the one below that is the best fit for your research. If you are not sure, read the appropriate sections before making your selection.

☒ Life sciences ☐ Behavioural & social sciences ☐ Ecological, evolutionary & environmental sciences

For a reference copy of the document with all sections, see nature.com/documents/nr-reporting-summary-flat.pdf

Life sciences study design

All studies must disclose on these points even when the disclosure is negative.

Sample size	32,552,534 people. We included all people vaccinated in England in the study period.
Data exclusions	We excluded people under age 16 (since vaccination was not recommended for this age group) or those who received Moderna vaccine (numbers too small)
Replication	We replicated our main findings using a Scottish population of 5.4 million people and obtained similar results.
Randomization	N/A. We conducted an observational study.
Blinding	N/A. We conducted an observational study.

Reporting for specific materials, systems and methods

We require information from authors about some types of materials, experimental systems and methods used in many studies. Here, indicate whether each material, system or method listed is relevant to your study. If you are not sure if a list item applies to your research, read the appropriate section before selecting a response.

Materials & experimental systems

n/a	Involved in the study	
☒	☐	Antibodies
☒	☐	Eukaryotic cell lines
☒	☐	Palaeontology and archaeology
☒	☐	Animals and other organisms
☐	☒	Human research participants
☒	☐	Clinical data
☒	☐	Dual use research of concern

Methods

n/a	Involved in the study	
☒	☐	ChIP-seq
☒	☐	Flow cytometry
☒	☐	MRI-based neuroimaging

Human research participants

Policy information about studies involving human research participants

Population characteristics	Patients aged 16+ vaccinated with ChAdOc1nCoV-19 and BNT162b2 and having admitted to hospital with outcome of interest in the study period (1-12-2020 to 31-05-2021) in England. There were 16,759,298 women and 14,219,053 men in the population.
Recruitment	We used data collected from National Immunisation Management System (NIMS) and Hospital Episode Statistics (HES) in England
Ethics oversight	National Health Service Reserach Ethics Committee (NHS REC) approval was obtained from East Midlands-Derby Reserach Ethics Committee [reference 04/03/2021].

Note that full information on the approval of the study protocol must also be provided in the manuscript.
